# Redox-Regulated Iron Metabolism and Ferroptosis in Ovarian Cancer: Molecular Insights and Therapeutic Opportunities

**DOI:** 10.3390/antiox13070791

**Published:** 2024-06-28

**Authors:** Dan Liu, Zewen Hu, Jinzhi Lu, Cunjian Yi

**Affiliations:** 1 Department of Obstetrics and Gynecology, The First Affiliated Hospital, Yangtze University, Jingzhou 434000, China; moon18390822196@yeah.net (D.L.); 2021710942@yangtzeu.edu.cn (Z.H.); 2 Hubei Provincial Clinical Research Center for Personalized Diagnosis and Treatment of Cancer, Jingzhou 434000, China; 3 Department of Laboratory Medicine, The First Affiliated Hospital, Yangtze University, Jingzhou 434000, China

**Keywords:** redox, iron metabolism, ferroptosis, ovarian cancer, tumor immune microenvironment, glycolysis

## Abstract

Ovarian cancer (OC), known for its lethality and resistance to chemotherapy, is closely associated with iron metabolism and ferroptosis—an iron-dependent cell death process, distinct from both autophagy and apoptosis. Emerging evidence suggests that dysregulation of iron metabolism could play a crucial role in OC by inducing an imbalance in the redox system, which leads to ferroptosis, offering a novel therapeutic approach. This review examines how disruptions in iron metabolism, which affect redox balance, impact OC progression, focusing on its essential cellular functions and potential as a therapeutic target. It highlights the molecular interplay, including the role of non-coding RNAs (ncRNAs), between iron metabolism and ferroptosis, and explores their interactions with key immune cells such as macrophages and T cells, as well as inflammation within the tumor microenvironment. The review also discusses how glycolysis-related iron metabolism influences ferroptosis via reactive oxygen species. Targeting these pathways, especially through agents that modulate iron metabolism and ferroptosis, presents promising therapeutic prospects. The review emphasizes the need for deeper insights into iron metabolism and ferroptosis within the redox-regulated system to enhance OC therapy and advocates for continued research into these mechanisms as potential strategies to combat OC.

## 1. Introduction

Ovarian cancer (OC), a common gynecological tumor primarily occurring post-menopause [1], rapidly spreads to pelvic and abdominal areas following its development. The hidden position of the ovaries, often obscured by abdominal fat, complicates early detection. Due to the lack of effective screening methods, many cases are often diagnosed at advanced stages, typically Stage III or IV. Post-diagnosis, treatment challenges include high rates of metastasis and chemoresistance, which reduce the effectiveness of therapies. Following systemic therapeutic interventions, the five-year survival rate stands at 50.8%, markedly lower than rates reported for other malignancies within the female reproductive system, such as breast and cervical cancer [2]. In response, the oncological research community is actively developing novel and more effective treatment modalities to enhance survival outcomes for patients afflicted with ovarian cancer.

Iron is a universally essential element, critical for various biological functions, including oxygen transport, DNA synthesis, and energy metabolism in all living organisms [3,4,5]. However, beyond its beneficial roles, iron, as an important component of redox reactions (involving reactive oxygen species (ROS) and antioxidants), can also contribute to adverse effects associated with pathological states, including cancer. Although the pathogenesis of ovarian cancer remains unclear, increasing evidence has indicated that iron plays a role in its development. For instance, Grzelak et al. found higher concentrations of ferrous iron (Fe^2+^) in malignant versus benign ovarian tumors [6]. Additionally, Ćwiertnia et al. observed that decreased expression of ferroportin and increased expression of transferrin in ovarian cancer tissues led to iron accumulation and enhanced tumor cell proliferation [7]. Interestingly, Torti et al. demonstrated that imbalances in iron metabolism can influence cancer growth. Targeting either iron excess, which promotes cancer cell death, or iron deficiency, which inhibits cancer growth, suggests a potential therapeutic strategy [8]. These findings underline the significant impact of iron metabolism on ovarian cancer’s progression and suggest that targeting iron metabolic imbalances might offer new therapeutic avenues.

Ferroptosis is a type of iron-dependent regulated cell death, marked by iron accumulation and lipid peroxidation that damages cell membranes [9]. Cancer cells rely heavily on iron for DNA synthesis, mitochondrial function, and cell cycle progression, driving their rapid growth. Consequently, they are vulnerable to disruptions in iron metabolism [10]. Excess iron promotes the formation of harmful ROS through the Fenton reaction, where Fe^2+^ catalyzes the conversion of H_2_O_2_ to hydroxyl radical (•OH), especially when the body’s antioxidant mechanisms are insufficient or compromised [11]. These elevated ROS levels lead to toxic lipid peroxidation, overwhelming the capacity of the body’s redox system and ultimately triggering ferroptosis [12]. Redox regulation, controlling redox reactions, is crucial for cellular stability and preventing diseases like cancer [11,12]. Battaglia et al. found that Erastin could induce ferroptosis in ovarian cancer cells (HEY line) only when a high concentration of the labile iron pool (LIP) was present. LIP refers to a small, dynamic fraction of intracellular iron that is loosely bound and chemically reactive. An iron chelator could counteract the ferroptosis induced by Erastin. In contrast, the COV318 ovarian cancer cell line appears to be resistant to Erastin-based treatment due to the low LIP concentration in these cells [13]. In addition, recent studies indicate that ovarian cancer cells upregulate anti-ferroptosis factors such as Solute Carrier Family 7 Member 11 (SLC7A11, also known as xCT), providing insights into its modulation and potential as a therapeutic target [14]. Therefore, summarizing the relationship between iron metabolism, ferroptosis, and ovarian cancer has become an urgent necessity.

In this review, we systematically examine the roles of iron metabolism and ferroptosis within the redox-regulated system during ovarian cancer development. By elucidating how these processes are connected to the disease’s pathogenesis, we highlight their critical importance and propose innovative treatment strategies that target these pathways. Additionally, we discuss current challenges and future research directions, emphasizing the need for a deeper understanding of iron metabolism and ferroptosis to advance ovarian cancer therapy. This exploration underlines the necessity of further investigating the body’s iron metabolic processes to promote the induction of ferroptosis in ovarian cancer.

## 2. Iron Metabolism in Ovarian Cancer

### 2.1. Normal Iron Cycle in the Human Body

**Figure 1:**
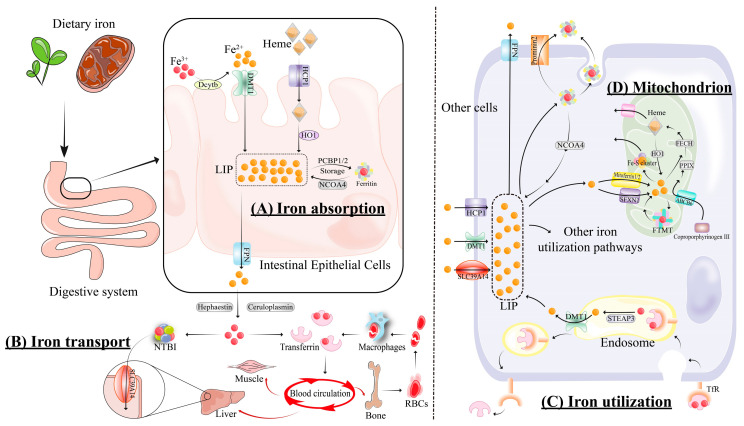
Normal iron cycle in the human body. (**A**) Iron is absorbed by intestinal epithelial cells: Although both animal liver/meat and vegetables contribute to the body’s iron intake through the digestive tract, they follow different absorption processes. The iron in meat is heme iron, which is absorbed directly into the intestinal epithelium via HCP1 and then broken down to Fe^2+^ by HO1. In contrast, iron in plants is non-heme iron and is first converted to Fe^2+^ by Dcytb before being absorbed by DMT1; (**B**) Iron transport in the circulation: Iron in the bloodstream is utilized by various organs to synthesize specific iron-containing components or is stored in specific proteins such as ferritin. For example, the liver uses iron to synthesize ferritin and hemosiderin, or stores it as inert iron in ferritin. Muscle tissue synthesizes myoglobin using iron, and the bone marrow produces red blood cells containing hemoglobin. Additionally, macrophages recover iron from damaged or aging cells, allowing it to re-enter the body’s iron cycle as Fe^3+^. However, circulating NTBI can be detrimental to organs. For instance, NTBI can enter hepatocytes via SLC39A14 and induce hepatic damage; (**C**) Iron utilization by other cells: Iron enters cells through transporters such as HCP1, DMT1, and SLC39A14. Transferrin-bound iron is recognized by TFR1 and endocytosed. In endosomes, acidic conditions release Fe^3+^, which is reduced to Fe^2+^ by STEAP3 and transported to the cytoplasm via DMT1. In the cytoplasm, Fe^2+^ joins the LIP, essential for cellular functions, including the synthesis of iron-containing proteins and enzymes. FPN mediates iron export, while Prominin 2 regulates iron homeostasis. NCOA4 facilitates ferritinophagy, releasing stored iron from ferritin into the cellular iron pool; (**D**) Iron is utilized in mitochondria: The mitochondrial membrane proteins mitoferrin 1/2 and SFXN1 transport Fe^2+^ from the cytoplasm into the mitochondria, where it participates in the synthesis of compounds such as Fe-S clusters and heme, or is stored in FTMT. Coproporphyrinogen III in the cytoplasm is converted to PPIX upon entry into the mitochondria via ABCB6. With the help of FECH, Fe^2+^ is incorporated into PPIX to form heme. These substances can either be stored in the mitochondria or transported to the cytosol to participate in various cellular reactions. Abbreviations: HCP1, heme carrier protein 1; HO1, heme oxygenase 1; Dcytb, duodenal cytochrome B; DMT1, divalent metal transporter 1; NTBI, non-transferrin-bound iron; FTMT, mitochondrial ferritin; SLC39A14, Solute Carrier Family 39 Member 14; STEAP3, the six-transmembrane epithelial antigen of the prostate 3; ABCB6, ATP Binding Cassette Subfamily B Member 6; PPIX, protoporphyrin IX; FECH, ferrochelatase; SFXN1, siderofexin 1; TFR1, transferrin receptor 1; FPN, Ferroportin; NCOA4, nuclear receptor coactivator 4.

Iron, an essential trace element, undergoes a complex and finely regulated cycle in the human body to ensure its proper distribution, utilization, and storage (Figure 1).

Dietary iron is available in two forms: heme and non-heme (or inorganic) iron [15]. In the intestines, non-heme iron is converted to Fe^2+^ by duodenal cytochrome B (Dcytb) and is absorbed through divalent metal transporter 1 (DMT1, also known as SLC11A2/DCT1/Nramp2) [16]. However, heme iron is absorbed through heme carrier protein 1 (HCP1) [17], and is then internalized and broken down to Fe^2+^ by heme oxygenase 1 (HO1) (Figure 1A).

Inside intestinal cells, iron can be stored in ferritin or transported into circulation as ferric iron (Fe^3+^) through the action of ferroportin (FPN), hephaestin, and ceruloplasmin [18]. In addition to being sourced from intestinal cells, iron in the body also comes from red blood cells and ferritin, and enters the circulation. Once in the bloodstream, transferrin distributes Fe^3+^ throughout the body. It is utilized by various organs to synthesize specific iron-containing components. For example, the liver uses iron to synthesize hemosiderin, muscle tissue uses it to produce myoglobin, and bone marrow uses it to generate red blood cells containing hemoglobin. The transferrin receptor (TfR) facilitates cellular iron uptake, which is then reduced to Fe^2+^ and joins the cytoplasmic LIP. However, in certain diseases, when circulating iron exceeds transferrin’s capacity, non-transferrin-bound iron (NTBI) forms. NTBI is absorbed by cells through non-transferrin iron transporters, including SLC39A14 (ZIP14), L-type/T-type calcium channels, DMT1, ZIP8, and TRPC6, potentially leading to associated pathologies [19] (Figure 1B).

The TfR on cells binds Fe^3+^-transferrin and internalizes it to form an endosome, where the low pH releases Fe^3+^ from transferrin. This iron is reduced to Fe^2+^ by the metal reductase of the six-transmembrane epithelial antigen of the prostate 3 (STEAP3), which enters the cytoplasm via DMT1, and is stored in ferritin, while transferrin is recycled or expelled (Figure 1C).

Cytosolic Fe^2+^ is transported into mitochondria via mitoferrin 1/2 and siderofexin (SFXN1), contributing to the synthesis of Fe-S clusters and heme, or stored in mitochondrial ferritin (FTMT) [20] (Figure 1D).

### 2.2. Regulation of Iron Homeostasis

Iron homeostasis involves distinct yet interconnected mechanisms at both the cellular and systemic levels. Cellular regulation encompasses mechanisms for iron uptake, storage, and release, while systemic regulation primarily involves hepcidin and its effects on iron absorption and distribution. These multilevel controls ensure proper iron distribution, fulfilling physiological demands while preventing iron overload and deficiency [21].

Iron uptake in cells primarily depends on the transferrin and TfR system, as well as the SLC39A14 channel, underscoring the cellular aspects of iron homeostasis. Many elements are crucial for maintaining iron homeostasis by modulating transferrin levels and the expression of TfR and SLC39A14 in cells. Follicle-stimulating hormone (FSH) influences transferrin levels [22], while retinoic acid (RA) [23] and the adipogenesis regulator (SREBP2) [24] promote transferrin synthesis. This is countered by CHOP/GADD153, which inhibits transferrin gene activation [25]. The expression of TfR, including TfR1 and TfR2 [26], is determined by cellular iron levels, health, hormones, and inflammation, and is boosted by conditions such as iron deficiency [27], hypoxia-inducible factor (HIF) [28], signal transducer and activator of transcription 5 (STAT5, a transcription factor activated by erythropoietin) [29], Ets-1 [30], nuclear factor erythroid 2-related factor 2 (Nrf2) [31], and GATA-1 [32]. Proteins such as sorting connexin 3 (Snx3) and Grab are vital for TfR recycling [33,34], while interleukin-2 (IL-2) stabilizes *TfR mRNA* against degradation [35]. The iron-responsive element (IRE)/iron regulatory protein (IRP) system governs TfR1 expression [36,37], EZH2 inhibitors enhance it by activating c-Myc transcription [38], and Holo-transferrin increases TfR2 levels [37]. SLC39A14, a metal transporter in the SLC39A family, assists in the uptake of NTBI, with potential regulation by the Wnt pathway through SRPK1 and SRSF1 [39]. Additionally, transcription factors AP-1, ATF4, and ATF6α support TfR expression [40].

Ferritin, a key form of cellular iron storage, can be degraded to release iron ions, which is crucial for iron homeostasis. The regulation of ferritin synthesis and degradation is essential for maintaining iron balance, and multiple factors are involved in this regulation. For instance, nuclear receptor coactivator 4 (NCOA4) promotes the autophagy of ferritin (ferritinophagy) under hypoxia, leading to the degradation of ferritin and the release of iron. Conversely, a decrease in NCOA4 levels results in increased ferritin accumulation [41]. The IRE/IRP system controls ferritin expression [42], while P53 [43] and NF-κB [44] boost its production. Additionally, the ubiquitin-proteasome system facilitates ferritin degradation [45]. These multiple mechanisms underscore the essential nature of ferritin regulation in cellular iron homeostasis.

Iron can exit cells via two primary pathways: through FPN as ions rejoining the systemic iron cycle, or through Prominin2 [46] or exocytosis as ferritin leaves the cell. FPN, the sole iron efflux channel in mammalian cells, is irreplaceable. Hepcidin, a liver-derived hormone, plays a crucial role in systemic iron regulation by inducing FPN phosphorylation and ubiquitination, leading to its degradation [47]. Elevated iron levels enhance hepcidin binding to FPN, reducing iron release, while decreased hepcidin levels reverse this effect [48]. FPN synthesis is also influenced by factors such as iron levels, inflammation, infection, hypoxia, and hemoglobin levels [49]. For instance, IL-6 upregulates hepcidin production during inflammation and infection, leading to increased hepcidin levels and decreased FPN expression [50]. Furthermore, Heme regulates the transcription of FPN1 through intermediates such as Bach1, Nrf2, and MARE/ARE [51]. Prominin2, involved in the iron stress response, facilitates the export of ferritin and is influenced by factors such as glutathione peroxidase 4 (GPX4) inhibitors [46], lipid peroxidation, and 4-hydroxynonenal (4HNE), with heat shock factor 1 (HSF1) acting as a key mediator [52]. Additionally, enzymes like HO1 participate in iron cycling and are vital for maintaining systemic iron balance.

### 2.3. Iron Metabolism in Ovarian Cancer

Iron plays a critical role in DNA synthesis, replication, repair, and translation, with its dysregulation linked to tumor development, including cancer [53,54]. Excessive iron exposure has been identified as a significant risk factor for ovarian cancer [55,56], impacting key processes like the Wnt signaling pathway, which is crucial for the OC’s onset and progression [57,58]. Additionally, iron has a negative influence on immune evasion by increasing iron levels, which reduces NO production and impairs macrophage and tumor cell-derived cytotoxic activity, ultimately protecting tumor cells from immune-mediated destruction [59].

Excessive and harmful ROS, such as •OH, produced by the catalysis of free iron, cause molecular damage, often referred to as “oxidative damage”. This damage encompasses DNA alterations, the activation of oncogenic pathways, such as those involving the Ras protein, and the inhibition of tumor suppressor pathways, including those involving P53 and PTEN, which can contribute to carcinogenesis. ROS also promote the MAPK pathway, facilitating the transformation of normal ovarian tissue into cancerous tissue [60]. In addition, ROS can also hydroxylate DNA residues to generate 8-hydroxy-2′-deoxyguanosine (8-OH-dG), which is highly mutagenic. Elevated 8-OH-dG levels are associated with poorer outcomes in patients with high-grade serous ovarian carcinoma (HGSOC) [61,62]. Iron-derived heme interacts with P53, leading to its degradation and increasing susceptibility to high-grade serous ovarian carcinoma [63,64].

Iron supports tumor cell proliferation, metabolism, and metastasis [65]. Several studies have demonstrated that reducing iron intake [66], lowering the concentration of transferrin [67] and its receptor (TfR) [68] in the body, or increasing the expression of FPN [69] on the cell membrane can inhibit tumor proliferation. Basuli et al. demonstrated that excessive iron promotes the proliferation and invasion of ovarian cancer cells [70]. Furthermore, iron can regulate the expression of HIF [71], which in turn promotes ovarian cancer development through various mechanisms, including the inhibition of P53, upregulation of IL-6, and regulation of non-coding RNA [72].

Iron metabolism’s role extends to chemotherapy resistance, with specific alterations in the iron transporter SLC40A1 affecting cisplatin resistance in ovarian cancer cells [73]. Upregulation of SLC40A1 decreases cisplatin resistance by exporting iron, reducing intracellular iron and oxidative stress (imbalance between free radicals and antioxidants). Conversely, the downregulation of SLC40A1 increases cisplatin resistance by accumulating iron, enhancing oxidative stress, and promoting survival pathways. Therefore, modulating iron levels to affect the redox system represents a potential strategy for overcoming treatment resistance.

### 2.4. The Impact of Iron Metabolism Pathology on Ovarian Cancer Progression

Epithelial ovarian cancer (EOC) is the most prevalent type of ovarian tumor [74], with the majority of cases in postmenopausal women falling into this category. This cancer is classified into five subtypes: high-grade serous ovarian carcinoma (HGSOC), endometrioid carcinoma (EC), clear cell carcinoma (CCC), mucinous carcinoma (MC), and low-grade serous ovarian carcinoma (LGSOC) [63]. The relationship between iron metabolism and the various subtypes of ovarian cancer may be complex.

HGSOC, often originating from the fallopian tubes, is typically diagnosed late. HGSOC has significantly more iron than normal ovarian tissue and LGSOC, indicating a connection between HGSOC and iron metabolism [75]. Additionally, Basuli’s team observed in HGSOC and ovarian cancer tumor-initiating cells (TICs) an increase in TfR1 and a decrease in FPN, resulting in higher iron levels and more cell proliferation. Lowering cellular iron levels can reduce ovarian cancer growth, particularly in TICs and HGSOC, but not in LGSOC [70]. Iron metabolism stability, managed through the transferrin-transferrin receptor 1 (Tf-TfR1) axis, is essential as its imbalance leads to DNA and gene instability, pushing toward HGSOC [76]. A study showed long-term iron exposure alters miRNA expression in fallopian tube cells, potentially aiding their conversion into HGSOC and promoting tumor growth and spread [77]. This highlights the role of iron in HGSOC development.

Endometrioid carcinoma (EC) and clear cell carcinoma (CCC), following HGSOC in prevalence among EOC, are believed to originate from atypical endometriosis. Despite arising from the same cell type, EC and CCC differ significantly in clinical outcomes; EC generally has a better prognosis, while CCC often resists chemotherapy [78], leading to poorer outcomes, especially when diagnosed at advanced stages. A detailed analysis of these subtypes revealed differences in iron metabolism gene expression, affecting iron storage and transport. In CCC, there is a notable reduction in the expression of FPN, lactoferrin, and transferrin, alongside an increase in ferritin light chain (FTL), indicating a high dependence on iron and highlighting iron’s critical role in CCC [79]. Yamaguchi et al.’s 2008 study linked the malignant transformation of endometriotic cysts into EC or CCC to prolonged exposure to high levels of free iron, finding that persistent oxidative stress induced genetic mutations. This transformation likely occurs when DNA damage outweighs repair capabilities [80].

Mucinous carcinoma (MC) of the ovary is rare, accounting for only 4% of cases, with the majority being diagnosed at stage I. Key genetic features of MC include mutations in KRAS, present in 40–50% of cases, and HER2/ERBB2 amplification, which occurs in 19% of cases [81]. Sano developed an scFv (single-chain variable fragment)—IONP (iron oxide nanoparticle) probe targeting ErbB2/HER2. Through MR (magnetic resonance) and photoacoustic imaging, this probe has been shown to be significantly absorbed by HER2-positive tumors, suggesting an increased iron concentration [82]. However, direct evidence linking altered iron metabolism to MC with HER2 amplification has yet to be established.

Low-grade serous ovarian carcinoma (LGSOC) is a rare type of EOC, which progresses slowly and is often resistant to chemotherapy. Like EC and CCC, LGSOC is linked to endometriosis, a condition where uterine-like tissue grows outside the uterus. Research shows that cysts associated with LGSOC, particularly those found in abnormal locations (ectopic cysts), contain significantly higher concentrations of free iron (100.9 mmol/L) than typical ovarian cysts (0.075 mmol/L) [80]. This elevated iron level could influence the behavior and development of the cancer.

Despite extensive data on common subtypes like HGSOC, EC, and CCC, there is a marked lack of research on rarer subtypes, creating gaps in our understanding of iron metabolism across different ovarian cancer subtypes. Additionally, the complexity of human metabolism, with its many interconnected pathways, suggests that disruptions in iron metabolism might influence broader metabolic processes, affecting ovarian cancer behavior and treatment outcomes.

## 3. Ferroptosis in Ovarian Cancer

### 3.1. Iron-Dependent Cell Death

Ferroptosis is characterized by an excessive accumulation of intracellular iron, which leads to toxic lipid peroxide build-up, redox system imbalance, and ultimately, cell death [83,84]. Morphologically, ferroptotic cells show significant mitochondrial changes, including reduced size, cristae loss, denser membranes, and fragmented outer membranes, while the nucleus remains largely unchanged [85]. Ferroptosis is frequently associated with inflammation. Compared to apoptosis, necroptosis, autophagy, and pyroptosis, ferroptosis has unique morphological features [83] (Table 1).

### 3.2. Mechanisms of Ferroptosis

**Figure 2:**
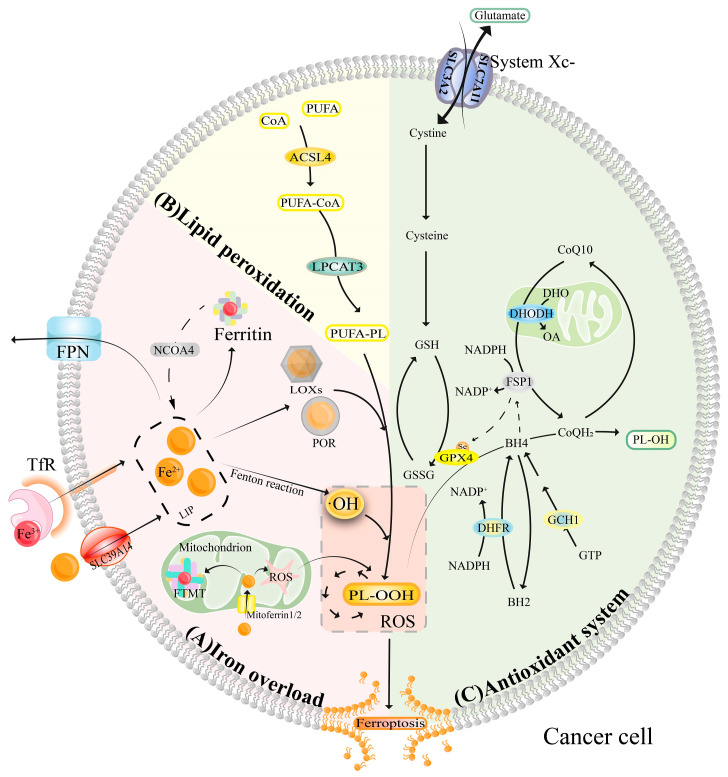
Ferroptosis: Regulated Cell Death Driven by Lipid Peroxides. (**A**) iron overload: iron overload occurs when excessive iron accumulates within the cell, both in the cytoplasm and mitochondria. This surplus iron is typically stored in ferritin to mitigate its potential toxicity. Excessive iron contributes to the LIP, which can participate in Fenton reactions, catalyzing the production of highly toxic •OH. These ROS, generated through various redox reactions, induce oxidative stress and are detrimental to cellular survival, leading to potential damage and dysfunction. (**B**) lipid peroxidation: Lipid peroxidation refers to the oxidative degradation of lipids, primarily targeting PUFAs. In the enzymatic pathway, enzymes such as LOXs and POR catalyze the oxidation of PUFAs, which have been incorporated into PUFA-PLs by ACSL4 and LPCAT3. This results in the formation of PL-OOH. In the non-enzymatic pathway, ROS, particularly •OH, directly react with PUFAs, leading to the generation of lipid radicals and subsequent propagation of oxidative damage. Both pathways contribute to the disruption of cellular membranes and can induce cell death, particularly through ferroptosis; (**C**) The antioxidant system includes four main pathways: The System Xc-GSH-GPX4 pathway, the FSP1-CoQ10 pathway, the DHODH-CoQH2 pathway, and the GCH1-BH4-DHFR pathway. The System Xc-GSH-GPX4 pathway primarily relies on GPX4 to oxidize GSH to GSSG and reduce PL-OOH to PL-OH. The remaining three pathways operate independently of GPX4. However, there are synergistic effects between the FSP1-CoQ10 pathway and the System Xc-GSH-GPX4 pathway, as well as between the GCH1-BH4-DHFR pathway and the FSP1-CoQ10 pathway. Abbreviations: LIP, labile iron pool; •OH, hydroxyl radical; ROS, reactive oxygen species; PUFAs, polyunsaturated fatty acids; LOXs, lipoxygenases; POR, cytochrome P450 oxidoreductase; PUFA-PL, polyunsaturated fatty acid-phospholipid; ACSL4, acyl-coenzyme A synthetase long-chain family member 4; LPCAT3, lysophosphatidylcholine acyltransferase 3; PL-OH, alcohol converted from the products of lipid peroxidation; PL-OOH, phospholipid hydroperoxides; GCH1, guanosine 5′-triphosphate (GTP) cyclohydrolase-1; GPX4, glutathione peroxidase 4; GSH, glutathione; GSSG, glutathione oxidized; BH2, dihydrobiopterin; BH4, tetrahydrobiopterin; CoA, coenzyme A; CoQ10, ubiquinone; CoQH2, ubiquinol; DHFR, dihydrofolate reductase; DHO, dihydroorotate; DHODH, dihydroorotate dehydrogenase; FPN, ferroportin; FSP1, ferroptosis suppressor protein 1; FTMT, mitochondrial ferritin; NADP+, the oxidized form of NADPH; NADPH, nicotinamide adenine dinucleotide phosphate; NCOA4, nuclear receptor coactivator 4; OA, orotate; TfR, transferrin receptor.

In contrast to other cell death pathways, ferroptosis, as the name suggests, is uniquely dependent on iron. This process involves at least three mechanisms: (a) iron overload, (b) lipid peroxidation, and (c) dysregulation of the antioxidant system (Figure 2).

In nature and in the human body, iron predominantly exists in two forms: ferrous and ferric ions. The unstable Fe^2+^ can catalyze the generation of highly toxic •OH through Fenton reactions [106]. Additionally, iron-containing enzymes such as lipoxygenases (LOXs) and cytochrome P450 oxidoreductase (POR) contribute to lipid peroxidation [107]. These mechanisms collectively drive the process known as organismal ferroptosis (Figure 2A).

The structural integrity of the cell membrane largely depends on lipids, particularly polyunsaturated fatty acids (PUFAs). Initially, the enzyme acyl-coenzyme A synthetase long-chain family member 4 (ACSL4) converts PUFAs into coenzyme A (CoA) derivatives. These are then transformed into polyunsaturated fatty acid-phospholipids (PUFA-PL) by lysophosphatidylcholine acyltransferase 3 (LPCAT3). PUFA-PLs are susceptible to oxidation through enzymatic (LOXs/POR) [108] and non-enzymatic (•OH, a strong oxidizing agent) [109] pathways, which produce phospholipid hydroperoxides (PL-OOH). Without timely conversion to phospholipid alcohol (PL-OH) by antioxidants, the accumulation of PL-OOH can lead to extensive lipid peroxidation, disruption of the antioxidant system in the organism, impaired membrane function, and ultimately, ferroptosis [108] (Figure 2B).

The body employs multiple pathways to generate antioxidants that eliminate lipid peroxides and protect against ferroptosis. A principal pathway involves the System Xc-, GSH, and GPX4 axis [110]. This involves the cysteine-glutamate antiporter, System Xc, on the cell membrane, which exchanges extracellular cystine for intracellular glutamate (mediated by SLC7A11 and SLC3A2). Inside the cell, cystine is converted to cysteine to synthesize glutathione (GSH). GPX4 then converts PL-OOH into the less harmful PL-OH by regulating redox reactions, thus mitigating damage and preventing ferroptosis. Inhibition of this pathway has been shown to induce ferroptosis in drug-resistant tumors [111]. Independently, the ferroptosis suppressor protein 1 (FSP1)-CoQ10 pathway, located at the plasma membrane, also prevents ferroptosis. It uses nicotinamide adenine dinucleotide phosphate (NADPH) to reduce ubiquinone (CoQ10) to ubiquinol (CoQH2), converting harmful PL-OOH to PL-OH. Another independent pathway, the DHODH-CoQH2 pathway, involves dihydroorotate dehydrogenase (DHODH) converting dihydroorotate (DHO) to orotate (OA) while simultaneously reducing CoQ to CoQH2, countering ferroptosis. Lastly, the GCH1-BH4-DHFR pathway facilitates the conversion of PL-OOH to PL-OH through tetrahydrobiopterin (BH4) produced by guanosine 5′-triphosphate (GTP) cyclohydrolase-1(GCH1) [112]. Dihydrofolate reductase (DHFR) helps maintain the balance between BH4 and dihydrobiopterin (BH2), converting NADPH to the oxidized form of NADPH (NADP+) [113]. Overexpression of GCH1/BH4 boosts CoQ10 production, further blocking ferroptosis [114], with noted synergistic effects with the FSP1-CoQ10 pathway (Figure 2C).

### 3.3. Ferroptosis in Ovarian Cancer

Ferroptosis, as a novel treatment for ovarian cancer, remains in the experimental stages, confined to cell line and animal model studies, and has not yet progressed to clinical trials. However, it has demonstrated promising potential in the treatment of ovarian cancer and may significantly prolong patient survival. Significant breakthroughs have been made in researching ferroptosis across various subtypes of ovarian cancer.

A 2019 report on ovarian cancer introduced Erastin, a ferroptosis inducer, which countered docetaxel resistance mediated by ABCB1 (a pathway promoting drug efflux from cells) [115] and promoted ferroptosis in ovarian cancer cells. A 2022 study by Wu et al. found that ferroptosis inducers targeting SLC7A11 enhanced the effectiveness of PARP inhibitors in platinum-resistant EOC cells, offering a new treatment strategy [116]. Further investigations by Xuan’s team showed that the iron-containing enzymes, fatty acid desaturases SCD1 and FADS2, were overexpressed in metastatic ovarian cancer cells, protecting against oxidative stress and enhancing malignancy. When SCD1/FADS2 was inhibited in cancer cells, the iron binding capacity decreased and the concentration of the LIP increased, leading to the accumulation of ROS and lipid peroxidation; with GPX4 also being downregulated, this led to ferroptosis. These inhibitors also reduced cisplatin resistance and, when combined with cisplatin, eradicated peritoneal metastasis in EOC [117]. Additionally, Yang et al. modulated the TAZ-ANGPTL4-NOX2 axis to induce ferroptosis in drug-resistant cells [118], while Alarcon-Veleiro’s group demonstrated that ferroptosis inducers altered small extracellular vesicles (sEV) in ovarian cancer cells, triggering ferroptosis and suggesting a novel mechanism of action [119].

Ferroptosis has demonstrated varied effects across different EOC subtypes, supported by extensive research. For example, Basuli et al. found that ferroptosis inducers could kill TICs in the HGSOC animal model, inhibiting growth and metastasis [70]. Marjamaa et al. showed that Erastin reduces HGSOC cell activity, especially when combined with cisplatin, though its effects can be reversed by a ferroptosis inhibitor [120]. Studies have ranked the susceptibility of different cancers to ferroptosis, which confirmed the feasibility of targeting ferroptosis to treat the HGSOC genome-scale metabolic (GSM) model [121]. The tumor suppressor gene P53, which is mutated in 96% of HGSOC cases [122], leads to reduced antioxidant pathways by suppressing the expression of SLC7A11, thereby promoting ferroptosis [123]. Significant differences in ferroptosis responsiveness between CCC and EC tumor and stromal cells have been observed. Research by Beddows indicated that CCC relies more heavily on iron, yet the high expression of GPX in CCC provides resistance against ROS-induced ferroptosis [79]. Thus, targeting GPX4 could be a novel treatment strategy for CCC, with promising initial results [124]. Furthermore, studies by Guo et al. revealed that inhibiting GSH metabolism raises ROS levels in CCC and HGSOC cells, while blocking ROS-induced ferroptosis reduces the chemosensitivity of CCC cells [125].

## 4. Molecular Crosstalk of Iron Metabolism and Ferroptosis

Molecular crosstalk between iron metabolism and ferroptosis plays a critical role in understanding the vulnerabilities of cancer cells. Iron, a vital element for cellular processes, becomes a double-edged sword when its regulation is disrupted. In cancer cells, aberrations in iron handling not only fuel uncontrolled growth by supporting essential metabolic activities but also set the stage for ferroptosis. Multiple factors are involved in the regulation of iron metabolism, and the same factors may affect ferroptosis through different pathways. This intricate interplay offers a promising avenue for targeted cancer therapies that exploit the unique iron dependency of tumor cells to induce cell death [126] (Table 2).

### 4.1. HO1

Heme oxygenase 1 (HO1) is critical in redox reactions, breaking down heme to release Fe^2+^, which can catalyze the production of ROS, leading to oxidative damage. In ovarian cancer cells, elevated HO1 levels enhance heme breakdown, increase ROS accumulation, and ultimately induce ferroptosis [141]. Zinc protoporphyrin IX (ZnPP), a HO1 inhibitor, has been shown to reverse Erastin-induced ferroptosis in these cells [173].

### 4.2. CP

Ceruloplasmin (CP), a liver-produced globulin and key member of the copper transporter family, plays a significant role in iron metabolism [174]. Working with ferroportin, CP oxidizes Fe^2+^ to Fe^3+^, which then binds to transferrin. This process lowers free Fe^2+^ concentrations, thereby reducing ROS production and inhibiting ferroptosis. Shang’s research on hepatocellular carcinoma indicates that reducing CP levels can induce ferroptosis, whereas increasing them can prevent it [153]. Similarly, a 2012 study by Huang et al. linked elevated CP levels with the development and progression of ovarian cancer [154].

### 4.3. IRPs/IREs

Iron regulatory proteins (IRPs), which are essential for cellular iron homeostasis, include IRP1 (also known as aconitase 1, ACO1) and IRP2. IRP1, an iron-sulfur protein [175], alternates between regulating gene expression by binding to iron-responsive elements (IREs) on the non-coding regions of mRNAs for TfR1 and ferritin, and acting as a cytoplasmic aconitase (ACO1) that helps maintain the reduced state of glutathione, a key antioxidant in living organisms [176]. IRP2, on the other hand, solely binds RNA and lacks catalytic activity. IRP activity is influenced by iron levels and other factors. Under iron-deficient conditions, both IRPs bind IREs to boost iron absorption by increasing TfR1 stability and decreasing ferritin levels, thus elevating free iron concentration [177]. In contrast, sufficient iron levels deactivate IRPs, leading to increased ferritin synthesis and TfR1 breakdown, reducing free iron. Environmental factors like oxygen levels and nitric oxide (NO) can modify IRP activity, particularly decreasing IRP2 activity under high iron conditions [161]. Recent studies indicate that IRP variations also impact other iron transporters like DMT1 and FPN1 [133]. For instance, in hepatocellular carcinoma, α-Enolase1 (ENO1) inhibits ferroptosis by reducing IRP1 expression [160], while in ovarian cancer, Takenaka et al. demonstrated that IRP2 overexpression under hypoxic conditions leads to increased iron uptake, which may promote carcinoma development [178].

### 4.4. HIF

Hypoxia-inducible factors (HIF), particularly HIF-1α and HIF-2α, are key regulators of cellular oxidation levels and play a pivotal role in tumor responses to hypoxia. Under hypoxic conditions, these factors significantly enhance tumor angiogenesis, accelerating growth and metastasis [179]. HIF-2α promotes the expression of Dcytb on the cell membrane [128], while HIF-1α increases the levels of DMT1, FPN1 [133] and TfR1 [164], thereby significantly raising the cellular iron content. Research indicates that differential HIF expression correlates with tumor invasion, worse prognosis, and advanced stages in EOC [180]. Studies on ovarian cancer cell lines HO-8910 and A2780 show that HIF-1α upregulates SLC2A12 under hypoxia, impacting GSH metabolism and inhibiting ferroptosis, thus promoting cancer progression [181]. Additionally, Duechler et al. discovered that HIF also plays a suppressive role in the immune microenvironment of ovarian cancer [182]. Overall, HIF critically affects ovarian cancer progression by modifying iron metabolism and ferroptosis.

### 4.5. Nrf2

Nuclear factor erythroid 2-related factor 2 (Nrf2) regulates oxidative reactions and stabilizes the intracellular environment [183]. It plays a crucial role in mitochondrial iron metabolism, essential for mitigating ferroptosis. Cytosolic Fe^2+^ is transported into mitochondria via mitoferrin 1 (SLC25A37) and mitoferrin 2 (SLC25A28), contributing to the synthesis of Fe-S clusters, heme, or stored in FTMT [20]. Disruptions in mitochondrial iron metabolism lead to increased ROS production and ferroptosis, exacerbated by the overexpression of mitoferrin 2 [184] and siderofexin (SFXN1) [185]. During heme synthesis, Nrf2 indirectly regulates intracellular iron metabolism. ABCB6, the only transporter for coproporphyrinogen III on the mitochondrial outer membrane [186], facilitates its entry into the mitochondria for conversion into protoporphyrin IX (PPIX) via oxidative reactions. FECH, the final enzyme in heme biosynthesis, incorporates Fe^2+^ into PPIX to form heme [187], which is then exported from the mitochondria. Nrf2 helps prevent iron accumulation in mitochondria by upregulating ABCB6 and FECH, thus preserving mitochondrial function. Sun et al. demonstrated that in liver cancer, the interaction between p62 (a crucial autophagosome formation protein) and Keap1 regulates Nrf2 expression. Inhibiting Nrf2 in cancer cells leads to decreased expression of NADPH Quinone Dehydrogenase 1 (NQO1), HO1, and ferritin heavy chain (FTH1), which promotes ferroptosis [140]. Additionally, Nrf2 influences the expression of TfR1 and FPN [106]. A 2015 study involving 90 patients with ovarian lesions showed that over half of the ovarian tumors expressed Nrf2, with its expression progressively increasing from normal tissue to benign, borderline, and malignant tumors [188]. This indicates a strong correlation between Nrf2 expression and ovarian tumor progression. Wang et al. found in ovarian cancer cell lines (CaoV3 and A2780) that the Nrf2/HO1/NQO1 pathway regulates cellular Fe^2+^ and ROS levels, impacting mitochondrial function, ferroptosis, and cell viability [189].

### 4.6. Non-Coding RNA, ncRNA

**Figure 3:**
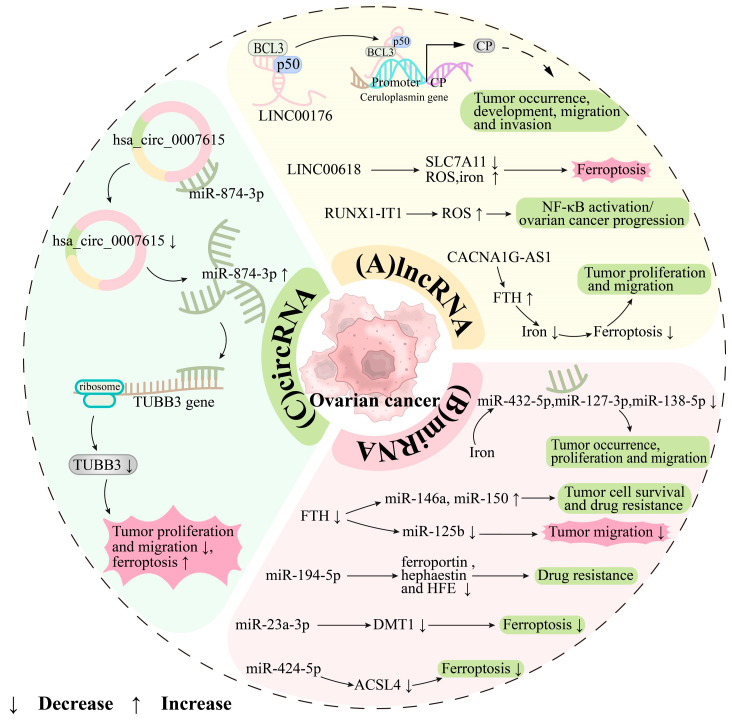
ncRNAs involved in the regulation of iron metabolism and ferroptosis in ovarian cancer. (**A**) lncRNA involved in the regulation of ovarian cancer: *LINC00176*, as a lncRNA, promotes the binding of BCL3 to p50, subsequently enhancing the activity of the *CP promoter*, which increases the expression of CP; (**B**) miRNA involved in the regulation of ovarian cancer: Abnormal iron metabolism can change the expression of miRNAs and affect the progress of cancer. In addition, the miRNAs can also target ovarian cancer by regulating iron metabolism; (**C**) circRNA involved in the regulation of ovarian cancer: circRNA is mainly involved in the regulation of cancer indirectly by affecting the action of miRNA on mRNA. Abbreviations: ncRNA, non-coding RNA; lncRNA, long noncoding RNA; BCL3, B-cell CLL/lymphoma 3; CP, ceruloplasmin; miRNA, microRNA; circRNA, circular RNA; SLC7A11, Solute Carrier Family 7 Member 11; ROS, reactive oxygen species; ACSL4, acyl-coenzyme A synthetase long-chain family member 4; FTH, ferritin heavy chain; DMT1, divalent metal transporter protein 1.

Genes can be classified into two categories based on their ability to be translated into proteins: coding RNA and non-coding RNA (ncRNA). The genome comprises both coding and non-coding regions, with the latter not producing proteins but playing a crucial role in regulating human survival and development. Remarkably, about 98.5% of the human genome consists of non-coding regions. These include genes for microRNA (miRNA), long non-coding RNA (lncRNA), and circular RNA (circRNA), which influence gene expression levels or patterns through various mechanisms, ultimately affecting the processes of life (Figure 3).

Long noncoding RNAs (lncRNAs) are RNA molecules consisting of more than 200 nucleotides that do not code for proteins, but are involved in the regulation of gene expression, cell differentiation, and various metabolic processes, including iron and redox metabolism. They are particularly significant in the development and progression of various cancers, including ovarian cancer. LncRNAs modulate iron metabolism, essential for DNA synthesis and cell proliferation in ovarian cancer. For example, *LINC00618* promotes ferroptosis in these cells by increasing ROS and iron levels and decreasing SLC7A11 expression [190]. In 2020, DAI et al. found that *LINC00176* enhances ovarian cancer growth, metastasis, and invasion by upregulating CP expression through interactions with B-cell CLL/lymphoma 3 (BCL3) and p50 [155]. The *lncRNA RUNX1-IT1* influences ROS balance, activating NF-κB and progressing the ovarian cancer [191]. Recently, Jin et al. (2023) reported that the *lncRNA CACNA1G-AS1* inhibits ferroptosis and supports cancer cell proliferation and migration by modulating FTH1 expression and iron availability [192]. Additionally, Feng et al. used bioinformatics to identify a set of ferroptosis and iron metabolism-related lncRNA (FIRL) signatures, including *AC138904.1*, *AP005205.2*, *AC007114.1*, *LINC00665*, *UBXN10-AS1*, *AC083880.1*, *LINC01558*, and *AL023583.1*, which could predict the prognosis and treatment response in ovarian cancer [193] (Figure 3A).

MicroRNA (miRNA) is a type of single-stranded, short non-coding RNA, around 20 nucleotides long, found in eukaryotes. It functions by binding to the 3′ non-coding region of target mRNA, either inhibiting translation or promoting degradation, which reduces protein production. The relationship between iron metabolism and ovarian cancer involves complex interactions. Abnormal iron metabolism can alter miRNA expression, impacting cancer progression. For example, Chhabra et al. observed that long-term iron exposure significantly changed the expression of specific miRNAs (e.g., *miR-432-5p* and *miR-127-3p* were downregulated by approximately 100-fold, and *miR-138-5p* by 16-fold), which ultimately promoted the transformation of fallopian tube secretory epithelial cells (FTSECs) into HGSOC and enhancing HGSOC cell growth and migration [77]. Lobello et al. found that silencing FTH in ovarian cancer changes miRNA levels (increasing *miR-146a* and *miR-150* while decreasing *miR-125b*), affecting cell survival, drug resistance, and migration [194]. Furthermore, miRNAs regulate iron metabolism. Wu et al. reported that *miR-194-5p* inhibits the expression of FPN, which is positively correlated with hephaestin and the homeostatic iron regulator (HFE), thereby inducing cisplatin resistance in ovarian cancer cells [195]. The *miR-23a-3p* affects iron metabolism by suppressing the expression of DMT1, inhibiting ferroptosis [131]. Moreover, Ma et al. demonstrated that *miR-424-5p* could attenuate ferroptosis in ovarian cancer by inhibiting ACSL4 [196] (Figure 3B).

Circular RNA (circRNA), discovered as early as 1976, has only recently caught the attention of the scientific community. These novel non-coding RNAs can act as miRNA sponges, absorbing and blocking miRNAs. This action inhibits the regulatory effects of miRNAs on their target mRNAs, thus affecting the expression levels of other genes and indirectly participating in epigenetic regulation. Recent experiments, including a study by Wei et al., have highlighted circRNAs’ roles in diseases such as ovarian cancer. Specifically, *hsa_circ_0007615* was found to be significantly elevated in EOC tissues compared to normal counterparts. Through comprehensive analysis, it emerged as an independent risk factor affecting the overall and recurrence-free survival rates in EOC patients. Silencing *hsa_circ_0007615* allowed *miR-874-3p* to target the *TUBB3 gene* effectively, inhibiting TUBB3 expression. This inhibition curtailed the proliferation and migration of ovarian cancer cells and promoted ferroptosis. Despite these insights, the link between circRNA, iron metabolism, and ferroptosis in ovarian cancer remains underexplored. Further investigation into this relationship could unveil new avenues for treating ovarian cancer [197] (Figure 3C).

## 5. Interactions between Iron Metabolism, Ferroptosis, Macrophages, T Cells, and Inflammation

**Figure 4:**
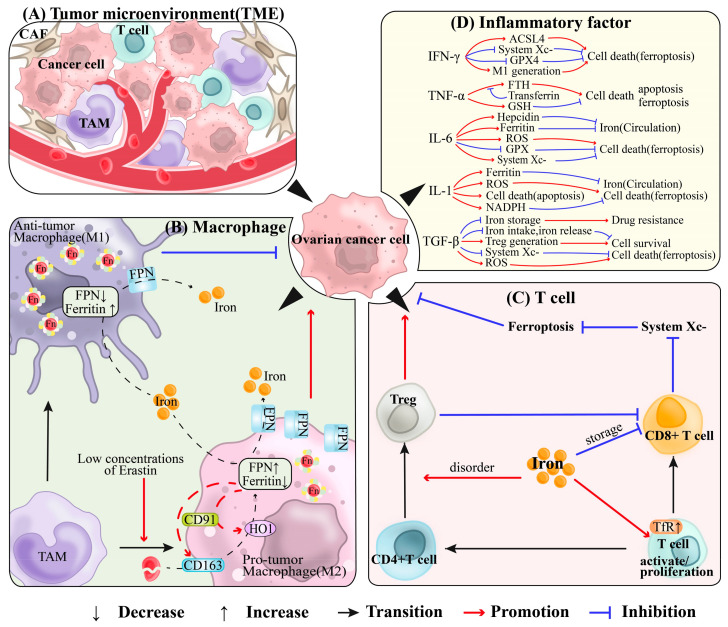
Factors involved in iron metabolism and ferroptosis in the tumor microenvironment of ovarian cancer. (**A**) TME: The progression of ovarian cancer is closely linked to the dysregulation of its TME, which includes cancer cells, CAFs, endothelial cells, macrophages, and T cells. CAFs release cytokines, influencing angiogenesis, immune responses, and immune escape. Tumor cells can convert stromal cells into CAFs. Endothelial cells regulate the entry of substances into tissues, crucial for transport, metastasis, and angiogenesis. (**B**) TAMs: M1 TAMs absorb iron via TfR1, store it as ferritin, and express low levels of FPN, limiting iron export and inhibiting tumor growth. Conversely, M2 TAMs acquire heme from senescent erythrocytes, producing Fe^2+^ via HO1. With high FPN and low ferritin expression, M2 TAMs release more iron, enhancing CD91 and CD163 expression. CD91 promotes HO1 production, while CD163 facilitates heme uptake, increasing environmental iron and promoting cancer development. (**C**) T Cells: Iron impacts various T cells. CD8+ T cells, crucial anti-tumor effectors, target and kill cancer cells, but often exhibit dysfunction or depletion in tumors. CD4+ T cells enhance the function of other immune cells, including CD8+ T cells. Tregs suppress anti-tumor immunity, supporting tumor survival. Iron promotes T cell activation and proliferation, induces the conversion of CD4+ T cells to Tregs, and negatively correlates with CD8+ T cells; (**D**) Inflammatory factors: Cytokines, as inflammatory factors, play complex roles in cancer. They can affect tumor progression through different pathways. IFN-γ induces lipid peroxidation (ACSL4), inhibits the antioxidant system (GPX4), and generates M1 macrophages, promoting ferroptosis. TNF-α inhibits ferroptosis via the antioxidant system (GSH) but induces ferroptosis in ovarian cancer cells by promoting FTH. IL-6, IL-1, and TGF-β exhibit context-dependent effects in the tumor microenvironment, potentially promoting or inhibiting ferroptosis. Abbreviations: TME, tumor microenvironment; CAF, tumor-associated fibroblasts; TAMs, tumor-associated macrophages; TfR1, transferrin receptor 1; FPN, ferroportin; FTH, ferritin heavy chain; HO1, heme oxygenase 1; GPX4, System Xc-/glutathione peroxidase 4; System Xc-, cystine-glutamate antiporter system Xc-; IFN-γ, interferon-γ; ACSL4, acyl-CoA synthetase long-chain family member 4; TNF-α, tumor necrosis factor-alpha; GSH, glutathione; IL-6, interleukin-6; TGF-β, transforming growth factor-β; ROS, reactive oxygen species; NADPH, nicotinamide adenine dinucleotide phosphate; Tregs, Regulatory T cells.

The tumor microenvironment (TME) consists of tumor cells with surrounding immune cells (e.g., macrophages and T cells), tumor-associated fibroblasts (CAF) and cytokines, and chemokines (Figure 4A). In the TME, changes in iron metabolism may affect ovarian cancer through multiple mechanisms.

### 5.1. Tumor-Associated Macrophage, TAM

Tumor-associated macrophages (TAMs), which make up over 50% of the tumor immune microenvironment (TIME), are critical to the biological behavior and prognosis of ovarian cancer. They are drawn to tumor sites by chemokines such as CCL2, CCL5, CXCL12, and CSF-1 (Figure 4B). TAMs are categorized into M1 and M2 types: M1 macrophages bolster anti-tumor immunity via pro-inflammatory responses and antigen presentation, while M2 macrophages, which secrete inhibitory cytokines and facilitate immune evasion, promote tumor growth and metastasis. However, it should be noted that the polarization of macrophages into M1 and M2 types is not always clear-cut, as there can be a spectrum of activation states based on different stimuli and tumor microenvironmental conditions. Larionova et al. reported that a higher TAM presence correlates with poorer prognosis in ovarian cancer. Specifically, a higher M1/M2 ratio is associated with better relapse-free survival (RFS) and overall survival (OS), while an increased density of CD163+ TAMs and a higher CD163/CD68 ratio are linked to worse RFS. The advanced stages are characterized by lower M1/M2 ratios and higher densities of CD163+ and CD68+ TAMs [198]. According to Akashi et al., M2 macrophages contribute to the transformation of epithelial cells in ovarian endometrioma into cancer cells. TAMs also affect iron storage and release, secrete cytokines such as IL-6, tumor necrosis factor-α (TNF-α), and interferon-γ (IFN-γ) that regulate iron metabolism, and enhance the invasiveness of ovarian cancer cells. Furthermore, they promote angiogenesis and chemoresistance, creating an immunosuppressive environment that supports tumor immune escape [199].

Research shows that M1 and M2 TAMs exhibit distinct behaviors in TIME due to differences in iron metabolism; M1 macrophages display high ferritin and low ferroportin levels, while M2 macrophages show the opposite [200]. As tumors evolve, they stimulate TAMs to transition to the M2 phenotype under the influence of T helper cell type 2 (Th2) cytokines like IL-4 and IL-13, enhancing tumor growth and metastasis [201]. M1 TAMs, activated by Th1 cytokines such as interferon-γ, target and destroy tumor cells by absorbing iron through TfR1 and storing it in ferritin, thus inhibiting tumor growth [202]. M2 TAMs, found in hypoxic zones, promote tumor proliferation by phagocytosing senescent erythrocytes, processing heme, and exporting iron via ferroportin. This iron release into TIME, along with molecules produced from decomposed heme, contributes to the tumor-promoting environment. This cycle, characterized by high ferroportin and low ferritin levels in M2 TAMs, is supported by the expression of CD91 and CD163, which aid in iron recycling and uptake, thus enhancing tumor spread [198,200]. Low concentrations of Erastin promote the migration of ferroptosis-resistant OC cells through the STAT3-mediated polarization of M2 macrophages [203]. Ji et al. found that TAMs promote the expression of SLC7A11 and GPX4 in the tumor microenvironment of ovarian cancer, thereby protecting endothelial cells from ferroptosis and supporting tumor development [204].

### 5.2. T Cell

T cells are crucial components of the immune system, playing a vital role in recognizing and eliminating tumor cells. The interaction between iron metabolism and T cell function in ovarian cancer represents a complex biological issue that involves immune regulation in the tumor microenvironment, iron availability, and tumor cell survival strategies (Figure 4C). Metabolism drives the differentiation, function, and fate of T cells. As an indispensable trace element, iron significantly impacts T cell activity in the immune system. For instance, T cell activation and proliferation are partly due to the upregulation of the TfR (also known as CD71) on their surface [205]. Abnormal iron metabolism can cause CD4+ T cells in tumors to differentiate into regulatory T (Treg) cells, aiding in the tumor’s immune evasion. Conversely, CD8+ T cells, indicators of iron overload severity, have their numbers inversely correlated with the body’s iron stores [206] and are pivotal in killing tumor cells. Wang et al. investigated various cell lines, including mouse ovarian cancer ID8. They discovered that CD8+ T cells can release IFN-γ to downregulate SLC3A2 and SLC7A11, components of System XC-, inhibiting cystine uptake by tumor cells. This action promotes lipid peroxidation and ferroptosis in tumor cells [207].

### 5.3. Inflammation

Inflammation significantly influences the fate of various cell components within the TME, and is intricately linked to iron metabolism. Inflammatory conditions within the TME prompt cells to release mediators such as cytokines and chemokines. These mediators can affect tumor cell proliferation and invasion through multiple pathways, including the regulation of iron distribution (Figure 4D).

Interferon-γ (IFN-γ) is produced by T cells, natural killer cells (NK cells), Treg cells, and B cells within the TME. Wall et al. showed that IFN-γ induces apoptosis in EOC cells in both in vivo and in vitro [208]. Lin et al. found that IFN-γ from CD8+ T cells binds to its receptor on cancer cells, reducing GPX4 and other antioxidants, promoting lipid peroxidation via ACSL4, and inducing ferroptosis [209]. Additionally, IFN-γ activates M1 macrophages, increases iron retention, and reduces its release, thereby inhibiting iron uptake by tumor cells and enhancing anti-tumor activity [201,202].

Tumor necrosis factor-α (TNF-α) is the earliest and most critical inflammatory mediator in the inflammatory response process, possessing cytotoxic effects on tumor cells. Fassl et al. demonstrated that TNF-α induces apoptosis in ovarian cancer cells by affecting FTH in both the human N.1/HTB77 ovarian cancer cell line and primary human ovarian cancer cells. Supplementation with transferrin could reverse this trend [210]. The TNF signaling pathway promotes GSH synthesis to protect cells from ferroptosis in a collagen-induced arthritis (CIA) mouse model [211].

Interleukin-6 (IL-6) is a multifunctional cytokine that significantly influences the development of ovarian cancer through complex pathways. Macciò et al. found that in advanced EOC (stages III/IV), pro-inflammatory cytokines (including IL-6) contribute to altered iron metabolism. This is marked by decreased red blood cell counts, reticulocyte counts, mean corpuscular volume (MCV), and serum iron, along with increased ferritin levels, leading to reduced circulating iron and increased ROS [212]. Liuzzi et al. noted that IL-6 induces hepcidin production, which reduces iron absorption in the intestines and its release from macrophages, causing hypoferremia [143]. Moreover, a large study by Macciò et al. involving 888 cancer cases showed that IL-6 was positively correlated with hepcidin, ferritin, and ROS, and negatively correlated with antioxidants like glutathione peroxidase. This suggests that IL-6 might modify the bio-availability of iron in the tumor microenvironment, impacting ovarian cancer development [213]. However, IL-6 also enhances ovarian cancer cell proliferation, migration, invasion, and drug resistance while inhibiting apoptosis [214]. IL-6 may facilitate ovarian cancer progression through additional pathways, such as IL-6/JAK2/STAT3, IL-6/STAT3/HIF-1α, and mtDNA/toll-like receptor (TLR9)/NF-κB/IL-6 [215]. Li et al. explored how IL-6 facilitates tumor progression by inducing ferroptosis resistance in head and neck squamous cell carcinoma [216]. Further research is necessary to fully understand IL-6’s complex interactions with ovarian cancer and to explore its potential in developing new treatment strategies.

Interleukin-1 (IL-1), part of the IL-1 family including IL-1α, IL-1β, and IL-1Ra, plays a key role in immune system regulation and disease development. Macciò et al. reported that IL-1β disrupts iron metabolism in advanced EOC (stage III/IV), leading to reduced red blood cell and reticulocyte counts, lower mean corpuscular volume (MCV) and serum iron levels, and increased ferritin, thus decreasing circulating iron [212]. Wang et al. found that IL-1α enhances the cytotoxic effects of carboplatin on ovarian cancer cells using the OVCAR-3 cell line, both in vitro and in vivo [217]. Additionally, Roy et al. observed that high levels of IL-1β induce apoptosis in genetically compromised cells and increase ROS [218]. IL-1β stimulation promotes NADPH production in cancer cells, which subsequently maintains adequate Fe-S clusters and protects tumor cells from ferroptosis [219]. These findings suggest that the IL-1β has variable effects on tumors, either promoting or inhibiting their progression.

Transforming growth factor-β (TGF-β) is a peptide cytokine involved in cell proliferation, differentiation, apoptosis, and immune escape in ovarian cancer. In a comprehensive analysis of 1669 cases of serous ovarian cancer using various gene microarrays and databases, Wu et al. discovered that TGF-β1/β2 influences ovarian cancer cells by inhibiting the uptake of iron. This inhibition is achieved by decreasing the expression of genes encoding for iron uptake proteins (Dcytb, CYBRD1, DMT1, STEAP3, STEAP2, TfR, SLC25A37, SLC39A14, and PCBP1) and increasing the expression of the heme transporter protein SLC48A1. Furthermore, TGF-β inhibits the export of iron by reducing the expression of FPN and affects iron storage by decreasing ferritin light chain 1 (FLH1) expression, which impacts ovarian cancer development, progression, drug resistance, and prognosis [220]. Kim’s study demonstrated that TGF-β1 increases ROS levels and enhances lipid peroxidation in cancer cells, ultimately leading to ferroptosis [221]. These findings suggest that TGF-β may serve as a novel target for ovarian cancer treatment.

In the ovarian cancer microenvironment, the interaction between macrophages, T cells, and inflammatory processes significantly impacts iron metabolism, ferroptosis, and cancer progression. M1 macrophages release pro-inflammatory cytokines (e.g., IL-1, IL-6, IL-12, IL-23, and TNF-α) to enhance immune responses, while M2 macrophages emit immunosuppressive factors like IL-10 and TGF-β, reducing T cell activity [201]. These macrophages also serve as antigen-presenting cells, activating T cell responses. Moreover, T cells produce cytokines such as IFN−γ, which promotes M1 polarization and restricts iron availability, thereby inhibiting cancer cell proliferation. While the role of iron metabolism and ferroptosis in tumor biology is well recognized, its specific interactions within the ovarian cancer immune environment require further research. Effective treatment strategies may include boosting T cell antitumor responses, adjusting macrophage polarization, and using immune checkpoint inhibitors, iron metabolism modulators like iron chelators or ferroptosis inducers to combat ovarian cancer.

## 6. Glycolysis-Related Iron Metabolism and Ferroptosis

Glycolysis plays a crucial role in cancer cell energy metabolism, especially under the “Warburg effect” where cancer cells prefer glycolysis over mitochondrial oxidation despite sufficient oxygen. This preference is partly due to the overexpression of HIF-1α in tumor cells. On one hand, HIF-1α can inhibit oxidative phosphorylation by activating pyruvate dehydrogenase kinase 1 (PDK1) and inhibiting pyruvate dehydrogenase (PDH). On the other hand, when activated HIF-1α binds to HIF-1 regulatory elements (HRE) of its target genes, it upregulates the expression of nearly all glycolysis-related genes (e.g., the key enzyme hexokinase (HK), phosphoglucose isomerase (PGI), pyruvate kinase M2 (PKM2), and lactate dehydrogenase (LDH)), thereby enhancing glycolysis. Additionally, HIF-1α promotes the synthesis of glucose transporter 1 (GLUT1), which provides more glucose for the energy metabolism of cancer cells. This reprogramming leads to high glucose consumption and lactate production, supporting ovarian cancer traits such as tumorigenesis, invasion, and drug resistance, and influencing iron metabolism. Icard et al. noted that the Warburg effect enhances lactate production and decreases mitochondrial activity, This weakens the catalytic effect of Fe^2+^ on ROS, maintaining non-toxic ROS levels and triggering processes like inflammation, DNA damage, genomic instability, epithelial-mesenchymal transition (EMT), angiogenesis, and cancer cell spread, Beyond influencing ROS concentrations, the Warburg effect also affects cancer cell development through various means, such as alterations in ATP, CO_2_, H^+^, citrate levels, and more [222]. Yao et al. found that energy pathways such as glycolysis, the pentose phosphate pathway, and the tricarboxylic acid (TCA) cycle affect ferroptosis markers by altering NADPH, GSH, and ROS levels [223]. Anderson highlighted that proliferating tumor cells rapidly deplete nutrients like sugars, amino acids, and fatty acids (FAs), creating a nutrient-poor TME. However, through metabolic reprogramming (Warburg effect), tumor cells can survive under these conditions and outcompete immune cells for essential nutrients. The significant uptake of glucose by tumor cells, compared to immune cells, leads to a suppression of anti-tumor immunity. Thus, a variety of nutrients, including glucose, amino acids, and the trace element iron, are more readily utilized by cancer cells, contributing to their development [224].

**Figure 5:**
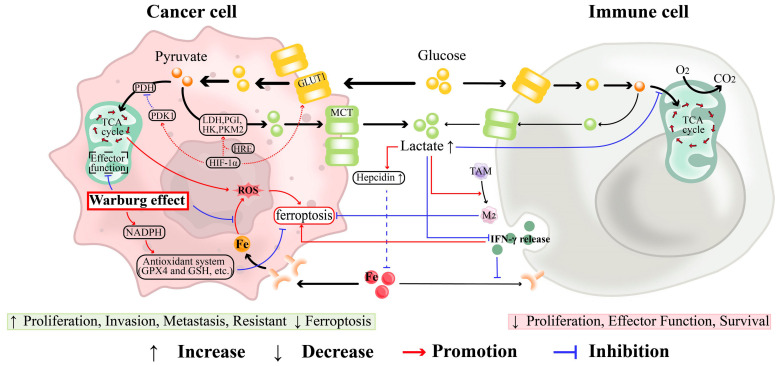
The Warburg effect, characterized by enhanced glycolysis and lactate production, plays a crucial role in inhibiting ferroptosis in tumor cells. Elevated lactate levels in the tumor microenvironment lead to increased hepcidin synthesis by the liver, reducing systemic iron levels. Lactate also promotes the formation of M2 TAMs, which can inhibit ferroptosis. Although IFN-γ can induce ferroptosis in tumor cells by promoting ROS production, lactate suppresses IFN-γ release from T cells and NK cells, thereby mitigating its pro-ferroptotic effects. Additionally, the Warburg effect suppresses iron-catalyzed ROS generation and enhances antioxidant defenses by increasing NADPH and GPX4/GSH levels, thus inhibiting ferroptosis. While iron overload in tumor cells can promote ROS production and subsequent ferroptosis, the overall impact of the Warburg effect and lactate is to reduce ROS levels and inhibit ferroptosis, thereby facilitating tumor cell survival. Additionally, this diagram illustrates the competitive absorption of nutritional factors between ovarian cancer cells and immune cells. The thicker black arrows denote higher rates of absorption and conversion. Abbreviations: TME, tumor microenvironment; TAMs, tumor-associated macrophages; GLUT1, glucose transporter 1; GSH, glutathione; GPX4, glutathione peroxidase 4; HIF, hypoxia-inducible factor; HK, hexokinase; HRE, HIF-1 regulatory elements; IFN-γ, interferon γ; LDH, lactic dehydrogenase; MCT, monocarboxylate transporter; NADPH, nicotinamide adenine dinucleotide phosphate; NK cells, natural killer cells; PDH, pyruvate dehydrogenase; PDK1, pyruvate dehydrogenase kinase 1; PGI, phosphoglucose isomerase; PKM2, pyruvate kinase M2; ROS, reactive oxygen species; TCA cycle, tricarboxylic acid cycle.

Lactate, a byproduct of glycolysis, not only affects iron metabolism and ferroptosis in ovarian cancer but also alters the tumor microenvironment. Mizumoto’s research found that lactate significantly increases hepcidin expression in acidic conditions, which regulates cellular iron [225]. Additionally, high lactic acid levels disrupt glycolysis, inhibiting IFN-γ release from T cells and NK cells, thus facilitating immune escape, inhibiting ferroptosis, and potentially increasing iron availability for tumor cells [224]. Jiang et al. showed that lactate drives macrophage polarization towards the M2 phenotype, enhancing iron utilization for cancer growth [226]. Therefore, the Warburg effect, characterized by enhanced glycolysis and lactate production, plays a crucial role in inhibiting ferroptosis in tumor cells. This metabolic reprogramming supports cancer cell survival and proliferation. Targeting these metabolic adaptations presents a promising avenue for cancer therapy (Figure 5).

## 7. Targeting Iron Metabolism and Ferroptosis in Ovarian Cancer Treatment

Iron metabolism and ferroptosis play critical roles in the pathophysiology of ovarian cancer. Current therapeutic strategies focus on inhibiting iron utilization by cancer cells, promoting the generation of ROS within these cells, and disrupting their antioxidant defense systems. In the following section, we will review the drugs under investigation for their potential applications in ovarian cancer, focusing on their mechanisms related to iron metabolism and ferroptosis. We will examine iron metabolism regulators, including those involved in absorption, storage, and transport, as well as multifunctional regulators. Furthermore, we will discuss the potential of iron chelators and delivery vectors (Table 3).

### 7.1. Iron Metabolism Regulators

#### 7.1.1. Iron Absorption Regulators

Although cells can absorb iron through various pathways, the absorption of iron by tumor cells primarily relies on TfR on the cell membrane surface. Regulating the expression or activity of these proteins can affect susceptibility to ferroptosis.

Ferristatin II: This compound reduces the levels of cell surface TfR1 and decreases cellular iron uptake, thereby lowering intracellular iron and mitigating ferroptosis. Cheng et al. investigated the relationship between ferristatin II, TfR1, and ferroptosis, and found that ferristatin II had a significant anti-ferroptotic effect in the HT-22 cell line. Further validation in in vivo experiments revealed that ferristatin II restored iron homeostasis by decreasing iron (Fe^3+^) levels and reversing the expression of iron homeostasis-related proteins, such as TfR1. Additionally, ferristatin II reversed the expression of lipid peroxidation genes and proteins, attenuating lipid peroxidation and exerting a protective effect [227].

Cobalt chloride (CoCl_2_) can activate IRPs by mimicking hypoxia. Petrova et al. observed changes in IRP expression after exposure to CoCl_2_. By affecting the expression of TfR1, FPN, and hepcidin, these changes ultimately led to altered iron homeostasis [228]. In addition, it has been demonstrated that CoCl_2_ exerts a protective effect on cells by inhibiting ferroptosis [229].

#### 7.1.2. Iron Storage Regulators

Bortezomib: As a proteasome inhibitor, it effectively increases NCOA4 levels by preventing proteasomal degradation, enhances iron autophagy, degrades ferritin, increases intracellular free Fe^2+^, and ultimately promotes ferroptosis [230].

Hemin, a natural iron supplement derived from animal blood, demonstrates potential as an anticancer agent. Hemin activates HO1, upregulates the expression of ferritin and FPN, and inhibits the expression of DMT1 and hepcidin [231], and finally increases labile iron (Fe^2+^) in cancer cells and enhances ferroptosis [232]. Additionally, hemin boosts IFN-γ production in CD8+ T cells, which further promotes ferroptosis in cancer cells, thereby inhibiting tumor growth [233].

#### 7.1.3. Iron Transport Regulators

Lapatinib, as a HER2 inhibitor, can enhance the sensitivity of ovarian cancer cells to carboplatin and significantly inhibit the growth of ovarian cancer [234]. An experiment on siramesine and lapatinib in 2016 proved that the combination of the two resulted in reduced FPN and increased transferrin expression, which led to an increase in the concentration of free iron in the cells, and then caused ROS accumulation and finally led to ferroptosis of cancer cells [235].

#### 7.1.4. Multifunctional Iron Metabolism Regulators

There are also drugs that are more specific and not only regulate multiple aspects of iron metabolism but may also affect other biological processes.

Sulforaphane: Found in cruciferous vegetables, this compound activates Nrf2 and can increase ferritin levels. Wang et al. found that sulforaphane upregulated ferritin and SLC7A11 levels by activating Nrf2, thereby inhibiting ferroptosis in cardiac cells in a mouse model of diabetic cardiomyopathy [236]. However, in ovarian cancer, although it inhibited ferroptosis, it promoted other types of cell death and ultimately inhibited cancer progression [237].

Artemisinins, derived from Artemisia annua, are known for their anti-malarial, anti-inflammatory, and anti-tumor effects. The compound (artesunate, ART) can induce ROS production and reduce proliferation in ovarian cancer cells [238]. Moreover, it can also promote cell death by enhancing the lysosomes’ ability to degrade ferritin and release iron [239].

Dihydroartemisinin (DHA) is involved in inducing iron overload and downregulating System Xc- [240]. Furthermore, artemisinin derivatives downregulate System Xc-, upregulate TfR expression, and affect the expression of various substances, including transferrin, ceruloplasmin, and lactoferrin, thereby influencing iron metabolism [241,242]. Additionally, DHA activates autophagy and ferritin degradation through the AMPK/mTOR/p70S6K pathway, increasing free Fe^2+^ and ROS levels [243]. These activities suggest that artemisinins could be effective ferroptosis inducers in ovarian cancer treatment.

Withaferin A (WA), derived from Withania somnifera (Ashwagandha), has historical uses in India for various conditions including epilepsy and diabetes, due to its therapeutic properties. Recent research has highlighted its potent anti-cancer effects [244] against breast [245], colon, cervical, ovarian [246], and lung cancers. Xing et al. demonstrated that WA enhances the labile iron (Fe^2+^) levels [247] in cells by activating HO1 in the iron metabolism process, and it directly targets and degrades GPX4 [248], leading to ferroptosis.

Shikonin, a natural compound from lithospermum, was used by Ni et al. in combination with cisplatin to treat OC cells resistant to cisplatin. The induction of ferroptosis by shikonin, achieved through increased ROS, Fe^2+^, and HO1 levels in cells and the downregulation of GPX4, was found to overcome cisplatin resistance [249].

Ferrous sulfate: Commonly used as an iron supplement for various forms of iron deficiency anemia, it increases the amount of iron that can be bound to transferrin. Sun et al. found that ferrous sulfate promotes the generation of ROS and lipid peroxidation, ultimately causing ferroptosis in E. coli by increasing the intracellular iron concentration [250].

BAY 11-7085 (BAY) is a well-known inhibitor of NF-kB. Majidinia found that BAY inhibits the proliferation of OVCAR-3 cells in ovarian cancer [251]. In addition, Chang et al. found that BAY induces overexpression of HO1 in cells via the Nrf2-SLC7A11-HO1 pathway, which increases the concentration of free Fe^2+^ and ultimately leads to the ferroptosis of cancer cells [252]. Ellinghaus et al. demonstrated that BAY exhibits anti-tumor activity by inhibiting mitochondrial complex I, which increases ROS levels and leads to a decrease in HIF-1 levels under hypoxic conditions [253].

The efficacy of FINO2 depends on iron levels, making it more potent in cancers with higher iron content [254]. Gaschler et al. demonstrated that FINO2 preferentially induces ferroptosis over other types of cell death. By directly oxidizing Fe^2+^, FINO2 indirectly inhibits the GPX4 enzyme, leading to extensive lipid peroxidation and ultimately triggering ferroptosis [255].

Iron nitroprusside (FeNP) has two negative effects on cells: it catalyzes highly toxic •OH through the Fenton reaction and consumes GPX4 to promote ferroptosis. In 2023, Asif’s team conducted in vitro and in vivo studies involving multiple cell lines—including ovarian cancer (SKOV3, A2780, and A2780cis), glioblastoma (U-87MG), and breast cancer (MDA-MB-231 and MCF-7)—and demonstrated that FeNP significantly increased ferroptosis in ovarian cancer cells compared to normal cells. By comparing normal liver cells with cancer cells, FeNP exhibited lower toxicity towards liver tissue and a stronger effect on cancer cells, indicating its potential for future use in the treatment of ovarian cancer [256]. FeNP fully exerted its NO donor and anti-tumor effects, including the generation of peroxynitrite which can damage tumor cells. Paradoxically, although FeNP promotes ferroptosis, it can also help tumor cells survive by increasing the level of iron within cells. This is due to the Warburg effect, unique to tumor cells, which inhibits the catalytic effect of iron on ROS. Feger’s work showed that elevated intracellular iron levels can rescue tumor cells from NO-mediated iron depletion, thereby preventing growth inhibition and apoptosis [257].

### 7.2. Iron Chelators

Iron chelators are compounds that bind to iron ions, forming stable complexes and thereby reducing free iron levels in the tumor cells.

Deferoxamine (DFO), a natural iron chelator used for treating iron overload, has shown promising effects in ovarian cancer treatment. Wang et al. tested DFO and cisplatin on ovarian cancer cell lines, finding that DFO not only inhibits cancer stem cells but also enhances cisplatin efficacy, improving chemotherapy resistance and overall survival [258]. Similarly, Brard’s team demonstrated that both DFO and diethylenetriamine pentaacetic acid (DTPA) exert cytotoxic effects on ovarian cancer cells, leading to cell cycle arrest and increased apoptosis in a dose- and time-dependent manner [259]. DFO also acts as a ferroptosis inhibitor, inhibiting ferroptosis in CCC [260].

Another iron chelator, deferiprone (DFP), also reduces intracellular iron levels, thereby inhibiting ferroptosis. Costa et al. demonstrated that DFP binds to nearly all iron in the body, preventing it from inducing the production of ROS. Consequently, DFP was categorized as a ferroptosis inhibitor and has been used in scientific research [248].

Deferasirox (DFX), an oral iron chelator, is used clinically to treat iron overload conditions and can inhibit ferroptosis by reducing the free iron pool within cells. In a mouse model of myocardial ischemia-reperfusion injury, Ishimaru et al. found that DFX reduced iron levels in the endoplasmic reticulum, ultimately inhibiting iron overload, lipid peroxidation, and ferroptosis in cardiomyocytes [261].

Quercetin: A flavonoid natural compound that might influence iron metabolism and is known for its inhibitory effects on several cancers. Horniblow et al. found that quercetin binds to intracellular iron, forming a complex. The increase in this iron complex in cells is distinct from the LIP, as the cells themselves exhibit signs of iron deficiency (increased TfR1 and decreased ferritin), and intracellular ROS are inhibited [262]. In addition, quercetin causes a decrease in System Xc-/GPX4 expression, promotes lipid peroxidation in gastric cancer cells, and induces ferroptosis [263].

Curcumin, derived from Curcuma longa, is known for its anti-inflammatory, antioxidant, and anti-proliferative effects. In ovarian cancer, it not only inhibits cell proliferation and invasion but also modulates ferroptosis and iron metabolism, contributing to its anti-tumor properties. Rainey et al. showed that curcumin acts as an iron chelator, similar to DFO, reducing ferritin levels and inducing autophagy and apoptosis, thereby inhibiting tumor growth [264]. Remarkably, Shi et al. found that the curcumin derivative NL01 induces ferroptosis in ovarian cancer cell lines by downregulating HCAR1/monocarboxylate transporter 1 (MCT1) and reducing GPX4 expression, which also inhibits tumor growth in xenograft models [265].

SK4, a novel iron chelator, demonstrates cytotoxic effects across various cancers, including breast, prostate, ovarian, and cervical, inducing cell death independently of P53 [266]. Abdelaal et al. showed that SK4 disrupts energy metabolism and causes iron deficiency, leading to abnormal mitochondrial function and cytotoxicity in ovarian cancer cell line SKOV3 and triple-negative breast cancer cell line MDA MB231 [267]. Similar to traditional iron chelators, SK4 inhibits ferroptosis and exerts a protective effect on cells [268].

Thus, these chelators have potential applications in anti-tumor therapy by limiting iron availability to tumor cells, which inhibits their proliferation. When used in conjunction with other treatment modalities, such as chemotherapy and radiotherapy, iron chelators may enhance therapeutic efficacy. Importantly, while iron chelators typically inhibit the onset of ferroptosis in cancer cells, including ovarian cancer, by reducing intracellular free iron levels and subsequent ROS production, this can also prevent the execution of ferroptosis. However, some chelators, such as quercetin and curcumin, can induce ferroptosis in ovarian cancer cell lines depending on their other biological functions, such as inhibiting antioxidant enzymes like GPX4. This dual functionality underscores the potential of iron chelators in comprehensive cancer treatment strategies, but also highlights the complexity of their roles in ferroptosis.

### 7.3. Delivery Vectors

Iron is absorbed by intestinal epithelial cells and distributed via transferrin, being taken up by cells through TfR on their surfaces. Although cancer cells may use alternative pathways, the transferrin-TfR pathway is the most significant. The construction of novel nanoparticles for drug delivery, utilizing both as targets, opens a new chapter in the field of drug delivery and represents a major breakthrough in tumor-targeted therapy.

Transferrin, the primary iron transport protein, demonstrates a higher affinity for cancer cells relative to normal cells, attributable to the increased iron demand of tumor cells. Utilizing this specificity, researchers have developed targeted therapies that employ transferrin as a drug delivery vector to selectively target cancer cells. For instance, Xu et al. engineered a metal-organic framework (MOF) by incorporating transferrin and piperlongumine (PL), a ferroptosis inducer that augments ROS production through the Fenton reaction, into the lipid layer. This innovation led to the creation of a dual-inductive nanosystem (transferrin-lipid layer of metal-organic framework connected with piperlongumine, Tf-LipoMOF@PL) capable of inducing both ferroptosis and pyroptosis. This system demonstrated significant anticancer efficacy in xenograft mouse models, highlighting its potential as a novel therapeutic approach for cancer [269].

TfR on the cell surface can bind to siderophores, facilitating the entry of extracellular iron into cells. Targeting TfR aims to inhibit the binding of transferrin to TfR, thereby reducing iron uptake by the cells, which in turn affects the proliferation and survival of tumor cells. Moreover, TfR1 is overexpressed on the surface of various cancer cell types, making it a promising therapeutic target. Tao et al. developed a novel therapeutic approach by combining chimeric antigen receptor-natural killer (CAR-NK) cell-derived exosomes (ExoCAR) with a nanobomb (Micelle) modified with a transferrin receptor binding peptide (T7), resulting in the formation of ExoCAR/T7@Micelle. This nanomaterial more effectively targets cancer cells, and upon internalization, the micelle, which promotes ROS amplification, is released at specific intracellular sites. This approach demonstrated a potent anti-tumor response in HER2-positive breast cancer brain metastasis (HER2+ BCBM) mouse models by disrupting the ferritin defense mechanism and inducing ferroptosis, all without significant side effects [270].

In cancer therapy, numerous drugs have been identified that promote ferroptosis in cancer cells by targeting iron metabolism through various pathways. However, many of the observed effects have been limited to cell lines and animal models, with a lack of comprehensive clinical trials. Treatments targeting iron metabolism and ferroptosis, particularly in ovarian cancer, remain experimental and have not yet become standard options due to challenges such as drug bioavailability, side effects, and tumor resistance. Further studies and clinical trials are needed to validate their safety and efficacy. Additionally, approved drugs must be carefully tailored to individual patients, as iron is essential for normal cellular functions, and interventions in iron metabolism can have serious side effects. In conclusion, while therapeutic strategies targeting iron metabolism are still in the research phase, ongoing research continues to explore this vital area of ferroptosis, potentially leading to the development of promising new treatments.

## 8. Conclusions and Future Directions

This review highlights the redox regulation of ferroptosis in the context of ovarian cancer. We discuss how the interplay between ROS-antioxidant systems and iron metabolism disorders presents novel therapeutic opportunities. The potential of targeting this balance to improve cancer treatment outcomes is explored, emphasizing the need for innovative strategies to manage redox systems and enhance ferroptosis.

Recent studies have underscored the critical link between ferroptosis, iron metabolism disorders, and ovarian cancer, suggesting intricate interactions that merit deeper exploration. While current research shows promise for targeting these pathways to enhance treatment efficacy and potentially improve immune function, most findings are still confined to the pre-clinical stages. Therefore, rigorous clinical trials are crucial to validate the safety and efficacy of these emerging therapies.

Moreover, while targeted therapies could complement existing treatments like chemotherapy and radiotherapy, balancing their potential benefits against risks to normal cells is imperative. The challenge of accurately predicting ferroptosis due to non-specific detection methods and variability among cancer subtypes highlights the need for more precise biomarkers.

Advancing our understanding of how iron metabolism influences ferroptosis—particularly the roles of the redox system and PUFA peroxidation—remains essential. This knowledge will help in designing targeted interventions that could mitigate drug resistance and suppress cancer progression. Given the pivotal role of ferroptosis in ovarian cancer pathology, continued research is vital to uncover new therapeutic targets and develop more effective treatments.

## Figures and Tables

**Table 1:** Different types of cell death.

	Morphologic Characteristics	Inflammation	Key Link/Factors	Regulation Mechanism	References
Ferroptosis	Mitochondria become smaller, cristae shrink or disappear, membrane density increases, outer membrane ruptures, and the nucleus is not significantly altered, but there are genetic changes.	Yes	Iron-dependence, lipid peroxidation	Fenton reaction, ROS regulation mechanism, System-Xc-GSH-GPX4 pathway, P53-related pathway, FSP1-CoQ10 pathway, Hippo pathway, GCH1–BH4 pathway, etc.	[9,83,84,85,86]
Apoptosis	Cellular crumpling, chromosome degradation, nuclear fragmentation, nucleolus deletion, vacuolization of cell membranes, and apoptotic vesicle formation.	No	caspase-3/7	Fas pathway, TNFR pathway, intrinsic/mitochondrial pathway, perforin/granzyme pathway, pro-apoptotic proteins, IAPs, death receptor pathway, death-inducing signal complex (DISC), Caspase 2/8/9/12, Ca^2+^, etc.	[87,88,89,90]
Necroptosis	Swelling of organelles, formation of necrosome, rupture of cell membranes, cytoplasm, and nuclei.	Yes	RIPK3	TNF pathway, caspase-8, RIPK1/3, MLKL, CYLD, CrmA, Fas ligand, toll-like receptors, LPS, etc.	[87,89,91,92,93,94,95,96]
Autophagy	Destruction of organelles, formation of large numbers of phagosomes, no changes in the nucleus or cell membrane.	No	mTOR	WIPI2, ULK complex, VPS34 complex I, ATG family, RAS, P53, Beclin-1, AMPK, NLRP3, Ca^2+^, etc.	[89,90,97,98,99,100,101,102,103,104]
Pyroptosis	Cells swell, the plasmalemma blisters, cell contents spill out, DNA breaks, inflammasome form, and organelle structures are preserved.	Yes	Gasdermin	C3, caspase 1/7, NLRP3, IL-1β, IL-18, etc.	[87,89,93,95,101,105]

Abbreviations: ROS, reactive oxygen species; GSH, glutathione; GPX4, glutathione peroxidase 4; FSP1, ferroptosis suppressor protein 1; CoQ10, ubiquinone; GCH1, guanosine 5′-triphosphate (GTP) cyclohydrolase-1; BH4, tetrahydrobiopterin; TNF, tumor necrosis factor; IL, interleukin.

**Table 2:** Factors affecting ferroptosis in iron metabolism.

Targeting Proteins in Iron Metabolism	Function	Implications for Ferroptosis	Adjustment Factors		Materials	References
			Induction	Inhibition		
Dcytb	Converts Fe^3+^ to Fe^2+^	Promote ferroptosis, inhibit ovarian cancer.	Fe low, HIF-2	high-fat diet (HFD)	* Animal model: C57BL/6 mice. * Clinical samples: blood samples from patients.	[127,128,129]
DMT1/SLC11A2/DCT1/Nramp2	Channel for Iron (Fe ^2+^) Entry	Promote ferroptosis, inhibit ovarian cancer.	Fe low, PCBP2, IRP 1, HIF-1α/2	miR-23a-3p	* Cell lines: astrocytes and neurons. * Animal model: mice. * Clinical samples: duodenal biopsy specimens from patients	[16,128,130,131,132,133]
HCP1/SLC46A1/PCFT	Channel for Heme	Promote ferroptosis, inhibit ovarian cancer.	O2 low, KLF 4, NRF 1, YY 1, CDX 2, HNF 4 α, Glucocorticoid (GC)	Cadmium (Cd)	* Cell lines: HeLa, CHO, HEK 293, * Animal model: C57/BL6 mice, rat	[17,134,135,136,137]
HO1	Converts Heme to Fe^2+^	Promote ferroptosis, inhibit ovarian cancer.	CISD 2, Nrf2, *miR-141*, PCBP2	Keap 1, Bach 1	* Cell lines: 293T (SCSP-502), HepG2, HepG2/5-FU, HL60 (TCHu 23), HT1080 (TCHu170), hepatocellular carcinoma cells. * Animal model: RAW264.7 mouse	[51,132,138,139,140,141]
FPN1/SLC40A1/Ireg1/MTP1	Channel for Iron (Fe^2+^) export	Inhibit ferroptosis, promote ovarian cancer.	Fe low, CISD 2, Nrf2, *miR-141*, PCBP2, HIF-1α, CD10-	Hepcidin, Keap 1, *miR-485-3b*, Bach 1, MARE/ARE, IL-6, IRP 1	* Cell lines: 293T (SCSP-502), HT1080 (TCHu170), HL60 (TCHu 23), HepG2, HepG2/5-FU, MCF-7, T-47D, HCC70, Hs-578T, BT-549, MDA-MB-361, hepatocellular carcinoma cells, astrocytes and neurons, MDA-MB-231, and so on. * Animal model: RAW264.7 mouse, IL-6 knockout mice, WT control mice of the C57BL/6. * Clinical samples: duodenal biopsy specimens from patients	[51,130,132,133,138,139,140,142,143,144]
Prominin2/PROM2	Channel for ferritin	Inhibit ferroptosis, promote ovarian cancer.	GPX4i, 4HNE, HSF1, BBOX 1-AS 1, *miR-326*, CTCF, RP 11 -89	miR-129-5p	* Cell lines: NCI H1975, S1, MCF10A, Hs578T, MDA-MB-231, SW640, SW860, SF295. * Clinical samples: non-small cell lung cancer and adjacent non-tumor tissues, bladder cancer, and adjacent non-tumor bladder mucosal tissues.	[46,52,145,146,147]
Hephaestin	Converts Fe^2+^ to Fe^3+^	Inhibit ferroptosis, promote ovarian cancer.	Fe low, CD10-, CDX 2	Cu low, HFE, G9a	* Cell lines: colorectal cancer cells, OVCAR3, TOV-21G, HT29, HeLa, human embryonic kidney 293, MCF-7, T47D, S1, SKBR3, HBL100, ZR-75-30, MDA-MB-231, MDA-MB-435, MDA-MB-468. * Animal model: C57BL/6J mice * Clinical samples: endometriosis-derived mesenchymal stem cells were isolated from primary human benign endometriosis deposits involving the ovary or fallopian tubes obtained from the surgical resection specimens of females	[144,148,149,150,151,152]
Ceruloplasmin (CP)	Converts Fe^2+^ to Fe^3+^	Inhibit ferroptosis, promote ovarian cancer.	*LINC00176*, BCL3, vitamin D	*miR-543*, COMMD10, Annona muricata leaf extract (AMLE)	* Cell lines: MHCC-97H, Huh7, HepG2, HCCLM3, Hep3B (human hepatocellular carcinoma cells), CaoV3, 3AO, skov3, HO8910, A2780 (ovarian cancer cell lines), CHO 1-15 (normal ovarian epithelial cell line), CNE2 (nasopharyngeal carcinoma). * Animal model: female BALB/c nude mice, rats. * Clinical samples: ascites samples from EOC patients, ovarian cancer and adjacent nontumor tissues, nasopharyngeal carcinoma tissues and cells, bone marrow or peripheral blood from blood malignancies/healthy people.	[153,154,155,156,157,158,159]
Transferrin	Transport Fe^3+^	Inhibit ferroptosis, promote ovarian cancer.	SREBP 2, FSH, RA	CHOP/GADD153	* Cell lines: Hep 3B (human hepatoma cell), Sertoli cells (rat). * Animal model: mouse models of tumorigenesis and metastasis * Clinical samples: peripheral blood samples from patients with metastatic melanoma	[22,23,24,25]
TfR (TfR1,TfR2)	transferrin receptor	Promote ferroptosis, inhibit ovarian cancer.	CISD 2, Nrf2, *miR-141*, Fe low, O2 high, NO, HIF-1α, Phosphorylation, IRPs, *miR-201*	Keap 1, NFS1, HFD	* Cell lines: HL60 (TCHu 23), HT1080 (TCHu170), 293T (SCSP-502), HepG2, HepG2/5-FU, MCF-7, BT-549, hepatocellular carcinoma cells, T-47D, A549, Hs-578T, MDA-MB-231, MDA-MB-361, HCC70, Friend leukemia cells. MCF10DCIS.com, 786-O, A498, SW900, NCI-H196, NCI-H322, NCI-H460, NCI-H647, NCI-H838, NCI-H2170, SK-MES-1, MDA-MB-231. * Animal model: SLC39A14(−/−), Hfe(−/−) and Hfe2(−/−) mice * Clinical samples: liver specimens from patients with chronic hepatitis B/C that underwent interferon therapy	[26,30,138,139,140,142,160,161,162,163,164]
SLC39A14/ZIP14	Transport NTBI	Promote ferroptosis, inhibit ovarian cancer.	AP-1, ATF4, ATF6 α, IL-6	Fe low, HFD	* Cell lines: Human embryonic kidney 293T. * Animal model: Hfe(−/−), Hfe2(−/−) and SLC39A14(−/−) mice, IL-6 knockout mice, WT control mice of the C57BL/6.	[40,143,163]
STEAP3	Converts Fe^3+^ to Fe^2+^	Promote ferroptosis, inhibit ovarian cancer.	Adipogenesis, Stress, P53 activation, Non-steroidal anti-inflammatory drugs, Fe low, infection	Capsaicin, LPS	Information from the review, no material.	[165]
Ferritin	Iron (Fe^3+^) storage	Inhibit ferroptosis, promote ovarian cancer.	PCBP 1/2, CISD 2, Nrf2, *miR-141*, TNF-α, NF-kB, NFS1, IL-1, IL-6	Keap 1, Fe low, O2 high, NO, Phosphorylation, IRPs, *miR-200b*, NCOA4	* Cell lines: HL60 (TCHu 23). HT1080 (TCHu170), 293T (SCSP-502), HepG2, HepG2/5-FU, MCF-7, T-47D, MDA-MB-361, BT-549, Hs-578T, A498, hepatocellular carcinoma cells, SW900, A549, MDA-MB-231, HCC70, 786-O, NCI-H196, NCI-H322, NCI-H460, NCI-H647, NCI-H838, NCI-H2170, SK-MES-1, MCF10DCIS.com, head and neck cancer cells. * Animal model: mouse tumor xenograft models	[132,138,139,140,142,160,161,162,166,167,168]
Mitochondrial ferritin (FTMT)	Mitochondrial iron storage	Inhibit ferroptosis, promote ovarian cancer.	SP1, CREB, Ying Yang 1 (YY1), HIF-1α, JNK, *miRNA-6862-5p*	GATA2, forkhead box protein A1 (FoxA1), CCAAT enhance-binding protein β (C/EBPβ), NCOA4	* Cell lines: HeLa, HT1080 fibrosarcoma cells, SH-SY5Y (neuroblastoma), fibroblast cell line, K562 (human erythroleukemic). * Animal model: transgenic drosophila.	[42,169,170,171,172]

Abbreviations: EOC, epithelial ovarian cancer; HIF, hypoxia-inducible factor; Dcytb, duodenal cytochrome B; IRP, iron regulatory protein; DMT1, divalent metal transporter protein 1; YY1, Ying Yang 1; HCP1, heme carrier protein 1, also called SLC46A1 or PCFT; Nrf2, nuclear factor erythroid 2-related factor 2; HO1, heme oxygenase 1; FPN1, ferroportin1, also called SLC40A1, Ireg1 or MTP1; GPX4i, glutathione peroxidase 4 inhibitors; 4HNE, 4-hydroxynonenal; HSF1, heat shock factor 1; HFE, homeostatic iron regulator; BCL3, B-cell CLL/lymphoma 3; FSH, follicle stimulating hormone; RA, retinoic acid; TfR, transferrin receptor; NTBI, non-transferrin-bound iron; STEAP3, the six-transmembrane epithelial antigen of the prostate 3; TNFα, tumor necrosis factor-α; NCOA4, nuclear receptor coactivator 4; IL, interleukin.

**Table 3:** Drugs Targeting Iron Metabolism and Ferroptosis for the Treatment of Ovarian Cancer.

Types of Drugs		Drugs	Mechanism	Materials	References
Iron metabolism regulators	Iron Absorption Regulators	Ferristatin II	Reducing TfR1 expression decreases intracellular iron levels, reverses lipid peroxidation-related gene and protein expression, alleviates lipid peroxidation, and inhibits ferroptosis.	* Cell lines: HT-22 cell line * Animal model: mouse traumatic brain injury model	[227]
		Cobalt chloride (CoCl_2_)	By affecting the expression of iron-regulatory proteins such as TfR1, FPN, and Hepcidin, iron homeostasis is altered, ultimately leading to the inhibition of ferroptosis.	* Animal model: suckling mice, contrast-induced nephropathy mouse	[228,229]
	Iron Storage Regulators	Bortezomib	Enhances iron autophagy, degrades ferritin, raises intracellular free Fe^2+^, and promotes ferroptosis by increasing NCOA4 levels.	* Cell lines: multiple myeloma cells	[230]
		Hemin	It activates HO1, upregulates ferritin and FPN, and inhibits DMT1 and hepcidin, leading to increased Fe^2+^ levels and ROS production in cancer cells. Additionally, CD8+ T cells are activated to secrete IFN-γ. Both mechanisms work together to promote ferroptosis.	* Cell lines: Jurkat-hTIGIT cells. * Animal models: CT26 tumor model and B16 tumor model, C57BL/6 J mice.	[231,232,233]
	Iron Transport Regulators	Lapatinib	It decreases ferroportin, increases transferrin, and accumulates ROS, leading to ferroptosis.	* Cell lines: MCF-7, ZR-75, MDA-MB-231, SKBR3 (breast cancer cells).	[234,235]
	Multifunctional Iron Metabolism Regulators	Sulforaphane	It raised the ferritin and SLC7A11 levels, inhibited ferroptosis, and inhibited the progress of ovarian cancer.	* Cell lines: A2780 and SKOV3 cells * Animal model: mice model of diabetic cardiomyopathy	[236,237]
		Artemisinins/Dihydroartemisinin (DHA)	Increasing ROS, increasing TfR expression, reducing ferritin and System Xc- concentration, thereby killing cancer cells and anti-proliferation.	* Cell lines: HeLa, HepG2, TSC2-WT/TSC2-KO mouse embryonic fibroblasts, primary liver cancer cell Hep3B (p53 null), Huh7 (p53 mutant), PLC/PRF/5 (p53 mutant), and so on.	[238,239,240,241,242,243]
		Withaferin A	It activates HO1, increases ROS, degrades GPX4, and promotes ferroptosis.	A variety of cell lines and animal models.	[244,245,246,247,248]
		Shikonin	Increased ROS, Fe^2+^ and HO1, downregulated GPX4, and induced ferroptosis	* Cell lines: A2780, SKOV3, OVCAR4, A2780(ovarian cancer cell), * Animal models: BALB/c nude mice.	[249]
		Ferrous sulfate	Increased the cell concentration of iron, promoted the transformation of ROS and lipid peroxidation, and led to ferroptosis.	* Cell lines: E. coli O157:H7	[250]
		BAY 11-7085 (BAY)	Elevated HO1, Fe^2+^, and ROS, reduced HIF-1, and induced ferroptosis.	* Cell lines: SKBR3, MCF-7, MDA-MB-468, MDA-MB-231(breast cancer cells), H460(non-small cell lung cancer cell).	[251,252,253]
		FINO2	Oxidized Fe^2+^, inhibited GPX 4, and induced ferroptosis.	* Cell lines: HT-1080 fibrosarcoma cells	[254,255]
		Iron nitroprusside (FeNP)	H_2_O_2_ and •OH are produced to induce ferroptosis.	* Cell lines: A2780, A2780cis, U-87 MG, MDA-MB-231, SKOV3, MCF-7 and MRC-5.	[256,257]
Iron chelators		Deferoxamine (DFO)	It improves chemoresistance, inhibits cancer stem cells, inhibits DNA synthesis, inhibits proliferation, induces apoptosis, and inhibits ferroptosis.	* Cell lines: SKOV-3, OVCAR-3 and NUTU-19 (rat) (ovarian cancer cell), RMG-1 and ES-2 (ovarian clear cell carcinoma).	[258,259,260]
		deferiprone (DFP)	It could bind to almost all the iron in the body and failed to further induce ROS production and inhibit ferroptosis.	Information from the review	[248]
		Deferasirox (DFX)	It reduces iron levels and inhibits iron overload, lipid peroxidation, and ferroptosis.	* Animal model: mice and rats model of myocardial ischemia-reperfusion injury	[261]
		Quercetin	It affects iron metabolism by increasing TfR1 expression and decreasing ferritin levels, inhibits ROS, and decreases the expression of System Xc- and GPX4, thereby promoting lipid peroxidation and inducing ferroptosis.	* Cell lines: RKO cells, AGS, HGC-27, MKN-7, MKN-45, SNU-1, and NCI–N87(gastric cancer cells), GES-1(gastric mucosal epithelial cell)	[262,263]
		Curcumin	It chelates iron similarly to DFO, induces autophagy and apoptosis by decreasing ferritin levels, and induces ferroptosis by decreasing HCAR1, MCT1, and GPX4, ultimately inhibiting tumor growth.	* Cell lines: Anglne, HO8910PM (ovarian cancer cells), Huh-7, T51B, RL-34 epithelial cells	[264,265]
		SK4	It decreases energy metabolism and produces cytotoxicity. It also inhibits ferroptosis and exerts a protective effect on cells.	* Cell lines: SKOV3 (ovarian cell line), MDA MB 231(triple negative breast cancer cell), LUHMES.	[266,267,268]
Delivery Vectors		Tf-LipoMof@PL	Transferrin was used as a carrier to deliver the drug, enhancing targeting ability, promoting ferroptosis and pyroptosis, and improving anti-tumor efficacy.	* Cell lines: 4T1 cells. * Animal model: 4T1 xenograft mice model	[269]
		ExoCAR/T7@Micelle	New nanoparticles were designed to specifically bind to TfR and promote ferroptosis, resulting in improved drug efficacy.	* Animal model: mice with orthotopic HER2-positive breast cancer brain metastasis.	[270]

Abbreviations: •OH, hydroxyl radical; DMT1, divalent metal transporter protein 1; FPN, ferroportin; GPX4, glutathione peroxidase 4; HIF, hypoxia-inducible factor; HO1, heme oxygenase 1; IFN-γ, interferon γ; MCT, monocarboxylate transporter; NCOA4, nuclear receptor coactivator 4; ROS, reactive oxygen species; TfR, transferrin receptor; Tf-LipoMof@PL, transferrin-lipid layer of metal-organic framework connected with piperlongumine; ExoCAR/T7@Micelle, a chimeric antigen receptor-natural killer cell-derived exosome combined with Micelle and modified with T7.

## Data Availability

Data available on request.

## Abbreviations

^•^OH	hydroxyl radical
4HNE	4-hydroxynonenal
8-OH-Dg	8-hydroxy-2′-deoxyguanosine
ACSL4	acyl-coenzyme A synthetase long-chain family member 4
AMLE	annona muricata leaf extract
ART	artesunate
BCL3	B-cell CLL/lymphoma 3
BH2	dihydrobiopterin
BH4	tetrahydrobiopterin
C/EBPβ	CCAAT enhance-binding protein β
CAF	tumor-associated fibroblasts
CAR-NK	chimeric antigen receptor-natural killer
CCC	clear cell carcinoma
Cd	cadmium
circRNA	circular RNA
CoA	coenzyme A
CoQ10	ubiquinone
CoQH2	ubiquinol
CP	ceruloplasmin
Dcytb	duodenal cytochrome B
DFO	deferoxamine
DFP	deferiprone
DFX	Deferasirox
DHA	dihydroartemisinin
DHFR	dihydrofolate reductase
DHO	dihydroorotate
DHODH	dihydroorotate dehydrogenase
DISC	death-inducing signal complex
DMT1	divalent metal transporter protein 1
DTPA	diethylenetriamine pentaacetic acid
EC	endometrioid carcinoma
EMT	epithelial-mesenchymal transition
ENO1	α-Enolase 1
EOC	epithelial ovarian cancer
ExoCAR	chimeric antigen receptor-natural killer cell-derived exosomes
ExoCAR/T7@Micelle	chimeric antigen receptor-natural killer cell-derived exosomes are combined with Micelle and modified with T7
FA	fatty acids
FeNP	iron nitroprusside
FoxA1	forkhead box protein A1
FPN	ferroportin, FPN1 is also called SLC40A1, Ireg1 or MTP1
FSH	follicle-stimulating hormone
FSP1	ferroptosis suppressor protein 1
FTH	ferritin heavy chain
FTL	ferritin light chain
FTMT	mitochondrial ferritin
FTSECs	fallopian tube secretory epithelial cells
GC	glucocorticoid
GCH1	guanosine 5′-triphosphate (GTP) cyclohydrolase-1
GLUT1	glucose transporter 1
GPX4	glutathione peroxidase 4
GPX4i	glutathione peroxidase 4 inhibitors
GSH	glutathione
GSSG	glutathione oxidized
HCP1	heme carrier protein 1, is also called SLC46A1 or PCFT
HER2+ BCBM	HER2-positive breast cancer brain metastasis
HFD	high-fat diet
HFE	homeostatic iron regulator
HGSOC	high-grade serous ovarian carcinoma
HIF	hypoxia-inducible factor
HK	hexokinase
HO1	heme oxygenase 1
HRE	HIF-1 regulatory elements
HSF1	heat-shock factor 1
IFN-γ	interferon γ
IL-1	interleukin-1
IL-2	Interleukin-2
IL-6	interleukin-6
IREs	iron-responsive elements
IRPs	iron regulatory proteins
LDH	lactic dehydrogenase
LGSOC	low-grade serous ovarian carcinoma
LIP	labile iron pool
lncRNA	long noncoding RNA
LOXs	lipoxygenases
LPCAT3	lysophosphatidylcholine acyltransferase 3
MC	mucinous carcinoma
MCT	monocarboxylate transporter
miRNA	microRNA
MOF	metal–organic framework
NADP+	the oxidized form of NADPH
NADPH	nicotinamide adenine dinucleotide phosphate
NCOA4	nuclear receptor coactivator 4
ncRNA	non-coding RNA
NK cells	natural killer cells
Nrf2	nuclear factor erythroid 2-related factor 2
NTBI	non-transferrin-bound iron
OA	orotate
OC	ovarian cancer
OS	overall survival
PDH	pyruvate dehydrogenase
PDK1	pyruvate dehydrogenase kinase 1
PGI	phosphoglucose isomerase
PKM2	pyruvate kinase M2
PL	piperlongumine
PL-OH	alcohol converted from the products of lipid peroxidation
PL-OOH	phospholipid hydroperoxides
POR	cytochrome P450 oxidoreductase
PPIX	protoporphyrin IX
PROM2	Prominin2
PUFA-PL	polyunsaturated fatty acid-phospholipid
PUFAs	polyunsaturated fatty acids
RA	retinoic acid
RFS	relapse-free survival
ROS	reactive oxygen species
sEV	small extracellular vesicles
Snx3	sorting connexin 3
STEAP3	the six-transmembrane epithelial antigen of the prostate 3
TAM	tumor-associated macrophage
TCA cycle	tricarboxylic acid cycle
Tf-LipoMof@PL	transferrin-lipid layer of metal-organic framework connected with piperlongumine
TfR	transferrin receptor
TGF-β	transforming growth factor-β
Th1	T helper cell type 1
Th2	T helper cell type 2
TIC	tumor-initiating cell
TIME	tumor immune microenvironment
TLR	toll-like receptor
TME	tumor microenvironment
TNF-α	tumor necrosis factor-α
WA	withaferin A
YY1	Ying Yang 1
ZnPP	Zinc protoporphyrin IX
